# High PGAM5 expression induces chemoresistance by enhancing Bcl-xL-mediated anti-apoptotic signaling and predicts poor prognosis in hepatocellular carcinoma patients

**DOI:** 10.1038/s41419-018-1017-8

**Published:** 2018-09-24

**Authors:** Jingjing Cheng, Dong Qian, Xiaofeng Ding, Tianqiang Song, Muyan Cai, Dan Xie, Yuwen Wang, Jinlin Zhao, Zhuang Liu, Zhiqiang Wu, Qingsong Pang, Li Zhu, Ping Wang, Xishan Hao, Zhiyong Yuan

**Affiliations:** 1 Department of radiotherapy, Tianjin Medical University Cancer Institute and Hospital, National Clinical Research Center for Cancer, Key Laboratory of Cancer Prevention and Therapy, Tianjin’s Clinical Research Center for Cancer, Tianjin, China; 2 Department of Pathology, State Key Laboratory of Oncology in South China, Cancer Center, Sun Yat-Sen University, Guangzhou, China; 3 Department of Hepatobiliary Surgery, Tianjin Medical University Cancer Institute and Hospital, National Clinical Research Center for Cancer, Key Laboratory of Cancer Prevention and Therapy, Tianjin’s Clinical Research Center for Cancer, Tianjin, China; 4 Department of Gastrointestinal Cancer Biology, Tianjin Medical University Cancer Institute and Hospital, National Clinical Research Center for Cancer, Key Laboratory of Cancer Prevention and Therapy, Tianjin’s Clinical Research Center for Cancer, Tianjin, China

## Abstract

Hepatocellular carcinoma (HCC) is the one of most common and deadly cancers, and is also highly resistant to conventional chemotherapy treatments. Mitochondrial phosphoglycerate mutase/protein phosphatase (PGAM5) regulates mitochondrial homeostasis and cell death, however, little is known about its roles in cancer. The aim of this study was to explore the clinical significance and potential biological functions of PGAM5 in hepatocellular carcinoma. For the first time, our results show that PGAM5 is significantly upregulated in HCC compared with corresponding adjacent noncancerous hepatic tissues and high PGAM5 expression is an independent predictor of reduced survival times in both univariate and multivariate analyses. Additionally, in vivo and in vitro studies showed that depleting PGAM5 expression inhibited tumor growth and increased the 5-fluorouracil sensitivity of HCC cells. Conversely, restoring PGAM5 expression in PGAM5-knockdown cells dramatically enhanced HCC cell resistance to 5-fluorouracil. Importantly, we demonstrated that the mechanism of 5-fluorouracil resistance conferred to HCC cells by PGAM5 was via inhibiting BAX- and cytochrome C-mediated apoptotic signaling by interacting and stabilizing Bcl-xL. Consistently, in the same cohorts of HCC patient tissues, Bcl-xL expression was positively correlated with PGAM5, and together predicted poor prognoses. In Conclusion, Our data highlight the molecular etiology and clinical significance of PGAM5 in HCC. Targeting the novel signaling pathway mediated by PGAM5/Bcl-xL may represent a new therapeutic strategy to improve the survival outcomes of HCC patients.

## Introduction

Worldwide, hepatocellular carcinoma (HCC) is one of the leading causes of cancer-related deaths;^1^ in China, HCC is the fourth most common cancer and the third leading cause of cancer-related death^2^. Despite diagnostic and therapeutic advancements, disease relapse limits HCC survival rates, as recurrent tumors respond poorly to chemotherapy^3^. Therefore, identifying new therapeutic targets for HCC is necessary, as, in contrast with other solid tumors such as breast, colon and melanoma, an important hallmark of HCC is the absence of clear oncogene addiction^4–6^.

Bcl-2 family proteins are key regulators of cell death that can either suppress (BAX, BAK, and Bim) or promote (Bcl-2, Bcl-xL, and Mcl-1) apoptosis^7–9^. In response to some lethal stimuli, the pro-apoptotic protein BAX translocates from the cytosol to the mitochondria to induce mitochondrial outer membrane permeability (MOMP)^10–12^. Upon MOMP, cytochrome C (cyt.C) is released into the cytosol where it cooperates with cytosolic factors to promote caspase activation, which leads to apoptotic cell death^13^. Conversely, the anti-apoptotic protein Bcl-xL neutralizes the pore-forming activity of BAX through an inhibitory interaction with BAX that prevents the generation of Ca^2+^ waves^14,15^.

Phosphoglycerate mutase/protein family member 5 (PGAM5) is an atypical mitochondrial serine/threonine phosphatase with homology to the phosphoglycerate mutase family, but lacking similar enzymatic function^16,17^. PGAM5 was first identified as a Bcl-xL interacting protein^16^, and subsequent reports have suggested that PGAM5 interacts with Keap1 in response to changes in mitochondrial function^18,19^. Recent studies have also shown that PGAM5 is critical for mitochondria homeostasis by regulating DRP1-mediated mitochondria fission^20,21^ and promoting mitophagy by interacting with FUNDC1^22,23^. Additionally, PGAM5 regulates multiple cell death pathways, including apoptosis and necrosis^24^. More recently, PGAM5 was shown to be a downstream anchor of the RIP1-RIP3-MLKL complex on mitochondria, and involved in necroptosis^24,25^. Conversely, BAX, Bcl-xL, and possibly other unidentified proteins are re-localized to the outer mitochondrial membrane by binding to PGAM5, which may regulate apoptosis by allowing the formation of a channel to release cyt.C^26,27,28^. However, the precise roles of PGAM5 in cell death regulation are still unclear.

Herein, we reported for the first time that elevated PGAM5 expression in HCC is associated with a poor prognostic phenotype. Knocking down PGAM5 in HCC cells inhibited cell viability and enhanced chemosensitivity. Additionally, we investigated the possible roles and molecular mechanisms of PGAM5 in HCC cell chemoresistance using in vitro and in vivo models.

## Materials and methods

### Patients and tissue specimens

HCC and corresponding adjacent noncancerous hepatic tissue samples were obtained with informed consent under Institutional Review Board-approved protocols. The samples were collected at the Tianjin Medical University Cancer Institute and Hospital (TMUCH, Tianjin, China) and the Cancer Center, Sun Yat-Sen University (SYSUCC, Guangzhou, China). The HCC cases selected were based on clear pathological diagnosis and follow-up data. All samples were formalin-fixed, paraffin-embedded and pathologically diagnosed. This study was approved by the Institute Research Ethics Committee of both TMUCH and SYSUCC. 178 patients with primary HCC, who underwent initial surgical resection between January 2010 and November 2011 in TMUCH, were used as testing cohort. The clinic-pathological characteristics of patients from testing cohort are summarized in Table S1.1. In parallel, we assessed another randomly collected independent validation cohort of 212 patients diagnosed with primary HCC, and underwent initial surgical treatment at SYSUCC between March 2003 and August 2006. The clinic-pathological characteristics from validation cohort are summarized in Table S1.2. Patients were selected for both the testing and validation cohorts only if they had been given a distinctive pathological diagnosis, were undergoing primary and curative resection, and had not received preoperative anticancer treatment. OS was defined as the time from surgery to the date of death from any cause or latest follow-up, while PFS was measured from the date of surgery until death, relapse or the cutoff date of 30 April 2016. Availability of patients’ resection tissues and follow-up data were also criteria for selection. Tumor differentiation was based on the criteria of the World Health Organization Classification of Tumors. Tumor stage was defined according to the American Joint Committee on Cancer/International Union Against Cancer Tumor/Node/Metastasis classification system.

### Cell lines

Five HCC cell lines (7721, 7402, LM6, Hep3B and HepG2) and one immortalized hepatic cell line (LO2) were obtained from ATCC (American Type Culture Collection, Manassas, VA, USA) or cell resource center of SIBS (Shanghai Institutes for Biological Sciences, Chinese Academy of Sciences, Shanghai, China). Cells were cultured less than 3 months after resuscitation and were maintained in Dulbecco’s modified Eagle medium (DMEM, Gibco Laboratories, Buffalo, Grand Island, NY, USA) with 10% (vol/vol) fetal bovine serum (Gibco Laboratories) at 37 °C in a 5% CO_2_ incubator. Cells were authenticated by short tandem repeat fingerprinting at Medicine Lab of Forensic Medicine Department of Sun Yat-sen University (Guanzhou, China).

### In vivo experiments

For in vivo study, 1 × 10^7^ mixed populations of HepG2 cells, stably knockdown of PGAM5 (shPGAM5), or the vector control, were injected subcutaneously into the flanks of BALB/C-nu/nu athymic nude female 4 weeks mice (six mice per group). When the tumor mass became palpable (about 200 mm^3^), mice in chemotherapy group were treated with 5-Fu (10 mg/kg, intraperitoneal injection, five times a week for 2 weeks). Using this model, we monitored the efficacy of 5-Fu treatment in the two groups. Tumor volume (V) was monitored by measuring the length (L) and width (W) of the tumor with calipers, and was calculated with the formula V = (L × W^2^) × 0.5. All procedures were carried out in accordance with the guidelines of the Laboratory Animal Ethics Committee of Tianjin Medical University Cancer Institute and Hospital. Over a 33 day period, Tumors were then excised and embedded in paraffin for hematoxylin and eosin staining, TUNEL assay and immunohistochemistry (IHC) analysis using anti-PGAM5 and Bcl-xL antibodies. All procedures were in accordance with the guidelines of the laboratory animal ethics committee of Sun Yat-Sen University.

### Tissue microarray (TMA)

TMAs were constructed in according with a previously described method^29^. Triplicate 0.6 mm diameter cylinders (two identical cylinders taken from intra-tumoural tissue and one cylinder from peritumoural tissue) were punched from representative areas of an individual donor tissue block, and re-embedded into a recipient paraffin block in a defined position, using a tissue array instrument (Beecher Instruments, Silver Spring, Maryland, USA)

### Immunohistochemistry (IHC)

The tissues were paraffin-embedded, and cut into 4-μm-thick sections, dewaxed using xylene, rehydrated through gradient ethanol, heated using the pressure cooker for antigen retrieval. Tissue slides were immersed in 3% hydrogen peroxide for 15 min, incubated with normal goat serum to block nonspecific binding, and then incubated overnight with anti-PGAM5 and anti-Bcl-xL antibody (1:200, Abcam, Cambridge, UK) at 4 °C, and then rinsed with PBS. Subsequently, the sections were incubated with a secondary antibody for 40 min at 37 °C, washed with PBS, and then stained with diaminobenzidine, followed by counter staining with hematoxylin. Finally, the sections were air-dried, dehydrated, and mounted.

### IHC evaluation

The expression of PGAM5 and Bcl-xL was assessed by three independent pathologists who were blinded to clinical follow-up data. If the two agreed with the scoring results, the value was selected. When there was a major disagreement, the pathologists reassessed the results until reaching a consensus. A staining index obtained as the intensity of positive staining (low, 1; moderate low, 2; moderate high, 3; strong, 4) and the proportion of immune-positive cells of interest (0 −25%, 1; 25–50%, 2; 50–75%, 3; 75–100%, 4) were calculated. The result of multiplying the scores of the two is the total score of the tissue (1, 2, 3, 4, 6, 8, 9, 12, 16).

### Western blot assay

Antibodies against PGAM5, Bcl-xL, BAX, Cyt.C, phospho-RIP3, and phospho-MLKL were purchased from Abcam (Abcam, Cambridge, UK). Antibodies against cleaved-caspase3, cleaved-PARP (poly (ADP-ribose) polymerase), ubiquitin and DYKDDDDK Tag were purchased form Cell Signaling Technology (Danvers, Massachusetts, USA). β-Actin (Santa Cruz Biotechnology, California, CA, USA) was used as normalized control. Cells were lysed in lysis buffer. Protein concentration was detected using a BCA kit. Western blotting was performed according to the standard procedures.

### CCK-8 assay

Cell proliferation/cell viability was estimated using a Cell Counting Kit-8 (CCK-8) assay. Cell suspensions (5000 cells/well) were added to 96-well plates in a volume of 200 ml/well. Each group was prepared with five parallel wells and incubated at 37 °C, 5% CO_2_, for indicated period. At the end of the culture period, 10 ul CCK-8 was added to each well. After 4 h incubation, the absorbance was measured with an enzyme calibrator at 450 nm and 630 nm, and the optical density values were measured.

### Plasmid construction, lentivirus production, and transduction

Plasmid containing the validated shRNAs targeted PGAM5 was cloned into the vectors pLLU2G (kindly provide by professor Dan Xie, Cancer Center, Sun Yet-Sen University) used in this study are derived from pLL3.7 and contain separate GFP and short hairpin RNA (shRNA) expression elements as well as required for lentiviral packging 42. The target sequences of PGAM5 for constructing lentiviral shRNA are 5′-AAGCTGGACCACTACAAAG-3′ (shRNA#1 or shPGAM5#1) and 5′-CCATAGAGACCACCGATAT-3′ (shRNA#2 or shPGAM5#2). For rescue experiments, a PGAM5 construct (mutations underlined: CCATAGAAACGACCGACAT) resistant to the shRNA#2 (CCATAGAGACCACCGATAT) was cloned into a pCDH cDNA expression lentivector (System Biosciences, Mountain View, CA, USA). The mutations do not affect the PGAM5 protein sequence. Then the lentiviral expression constructs (pCDH- PGAM5) and packaging plasmids mix were co-transfected into 293 cells to generate the recombinant lentivirus according to the manual.

### Annexin V–APC ⁄ propidium iodide (PI) flow cytometry apoptosis assay

Annexin V–APC and PI staining was performed to determine the percentage of cells undergoing apoptosis. The assay was carried out using the manufacturer’s protocol (BD Biosciences, Bedford, MA, USA). Each sample was then analyzed using a BD FACSCanto II flow cytometer (BD Biosciences) with annexin V on the horizontal axis and PI on the vertical axis.

### Clonogenic survival assay

Briefly, 7402 and HepG2 HCC cells were trypsinized, counted, and seeded for colony formation in 6-well plates with 100 cells per well. After incubation intervals of 14 days, colonies were stained with crystal violet and manually counted. Colonies consisting of 50 cells or more were scored, and five replicate wells containing over 10 colonies per well were counted for each treatment. Experiments were repeated three times.

### Immunoprecipitation

Immunoprecipitation was done by following the procedure described in earlier studies^30^. Cells (2 × 10^7^) were lysated and sonicated in a cell lysis buffer (20 mM Tris (pH 7.5), 150 mM NaCl, 1 mM EDTA, 1 mM EGTA, 1% Triton X-100, 2.5 mM sodium pyrophosphate, 10 mg/ml protease inhibitor cocktail) on ice. The antibodies used for immunoprecipitation goat anti- PGAM5 (Santa Cruz Biotechnology, Delaware, CA, USA), rabbit anti-BCL-xL, and rabbit anti-DYKDDDDK Tag (Danvers, Massachusetts, USA). Equal amounts of protein from whole cell lysate was incubated overnight with 1/100 volume of corresponding antibody at 4 °C with continuous rotation. Immune complexes were then precipitated by incubating them with 20 ul of proteinA/G agarose for 2 h at 4 °C. Immunoprecipitates were collected by centrifugation at 3000 rpm for 5 min followed by washing five times with a cell lysis buffer. After boiling in 20 ml 2 × SDS sample buffer, the samples were analyzed by western blotting using anti-PGAM5 and anti-BclxL antibodies. Western blot analysis followed the standard procedures and was repeated at least thrice for each protein tested.

### Immunofluorescence

Cells grown on coverslips were fixed in 4% paraformaldehyde, permeabilized with 1% Triton-100, and blocked with 10% normal goat serum. They were incubated with mitotracker (Beyotime Biotechnology, Shanghai, China), anti-PGAM5 (Abcam, Cambridge, UK), anti-BclxL (Abcam, Cambridge, UK), anti-BAX (Abcam, Cambridge, UK), anti-Cyt.C (Abcam, Cambridge, UK) and anti-γ-H2AX primary antibody (Cell Signaling, Danvers, MA, USA), for 1 h at 37 °C in a humid chamber, and then incubated with secondary antibodies for 1 h. Immunofluorescence images were captured using FV10-ASW viewer software (Olympus, Tokyo, Japan).

### TUNEL assay in situ

Apoptotic cells of the serial sections of xenograft tumors were determined by the terminal deoxyribonucleotidyl transferase (TDT)-mediated dUTP-digoxigenin nick end labeling (TUNEL) assay. Apoptotic cells were determined using the In Situ Cell Death Detection Kit POD (Roche Diagnostics, Basle, Switzerland), which quantitatively determines DNA fragmentation visualized with a fluorescence microscope. Immunofluorescence images were captured using ZEN viewer software (ZEISS, Oberkochen, Germany). Apoptotic indices were obtained by counting the percentage of TUNEL-positive cells.

### Statistical analysis

Statistical analysis was performed using SPSS 17.0 (SPSS Standard version, SPSS Inc., Chicago, IL, USA). The two-tailed Student’s *t*-test was used to analyze data expressed as means ± standard error. The chi-squared test was used to analyze the association between PGAM5 expression and clinicopathological features of HCC patients. Survival curves were analyzed using the Kaplan–Meier method. The significance test was analyzed using log-rank method. Univariate and multivariate survival analysis were analyzed using the Cox-regression model. *p* value < 0.05 was deemed significant.

## Results

### PGAM5 is frequently up-regulated in HCC

**Fig. 1:**
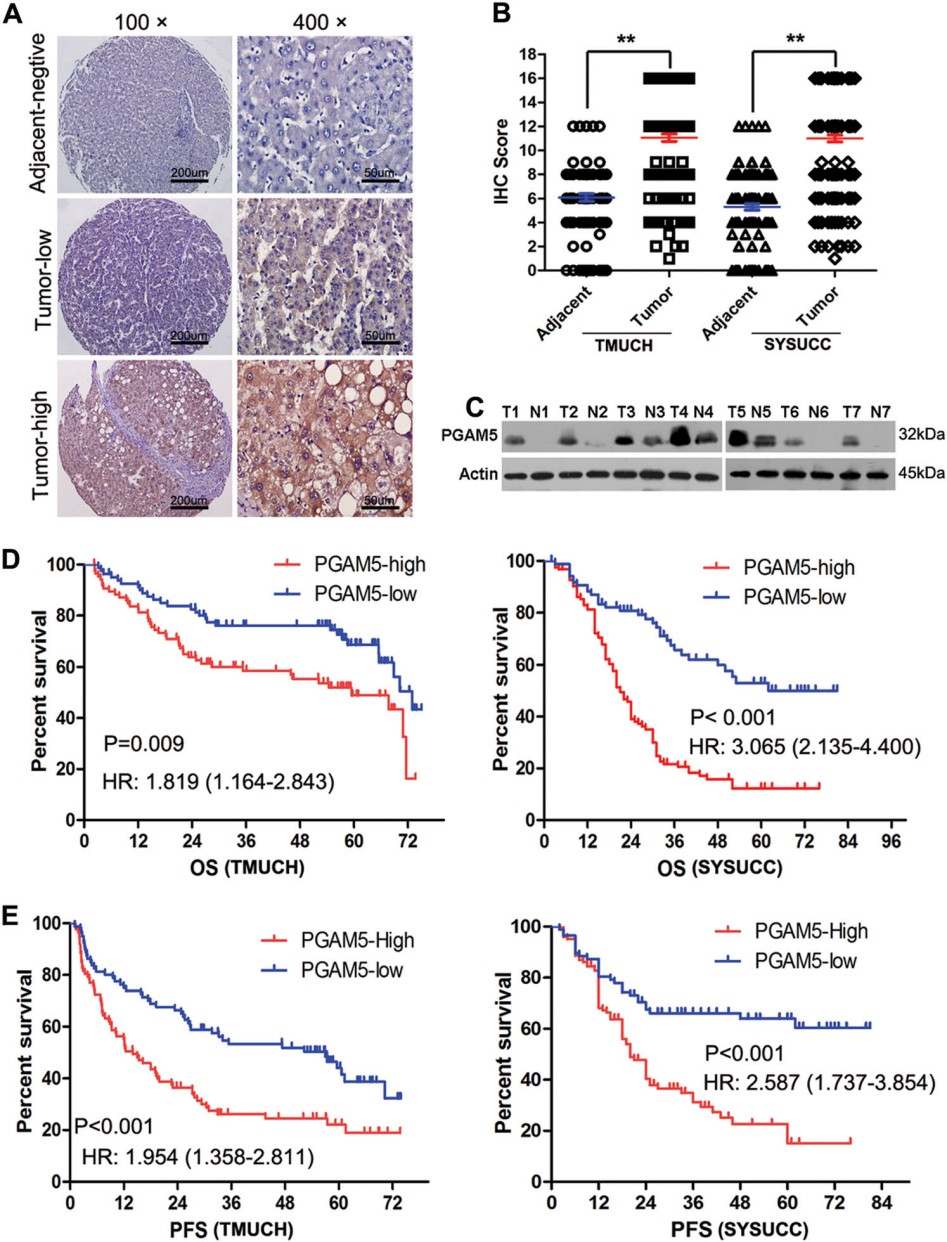
PGAM5 expression in HCC tissues and its prognostic significance in patients with HCC. **a** Representative immunohistochemistry staining of PGAM5 in HCC tissues and in adjacent non-neoplastic liver tissue (Original magnification: Left X100; Right X400). **b** PGAM5 was significantly up-regulated in HCC samples of both testing cohort (TMUCH, left, *p* < 0.01; Mann–Whitney test) and validation cohort (SYSUCC, right, *p* < 0.01; Mann Whitney test). **c** Western blotting showing higher PGAM5 levels in HCC fresh tissues than in paired adjacent non-neoplastic hepatic tissues in all 7 cases (T, hepatocellular carcinoma tissue; N, non-neoplastic hepatic tissues). **d**, **e** Kaplan-Meier survival analysis show that higher PGAM5 expression predicts poorer Overall Survival (**d**) and progression-Free survival (**e**) in 178 HCC patients of the testing cohort (left) and in 212 cases of the validation cohort(right)

No previous study has evaluated PGAM5 expression in HCC; thus, we detected PGAM5 protein by IHC in 178 HCC and 92 adjacent non-tumor tissues from TMUCH, and 212 HCC and 135 adjacent non-tumor tissues from SYSUCC as validation. Typical IHC images for the evaluation criteria were exhibited in Supplementary Fig.S1. According to the scoring criteria detailed above, PGAM5 was strongly up-regulated in HCC tissues compared with adjacent non-tumor tissues (Fig.1a, b, **p < 0.01). Additionally, western blotting was used to compare PGAM5 expression in 7-matched pairs of cancer and adjacent non-tumor liver tissue. Higher PGAM5 expression was found in all primary HCC tissues compared with the fresh adjacent non-neoplastic liver tissues (Fig. 1c).

### High PGAM5 expression in HCC patients is associated with poor prognoses

To address the clinical significance of overexpressed PGAM5 in HCC, correlations between PGAM5 and clinicopathological features of patients from both cohorts were examined. We subjected the PGAM5 IHC score to receiver operating characteristic curve analysis with respect to each clinicopathological variable. The corresponding area under the curve and cutoff scores for high PGAM5 expression are shown in Supplementary Fig.S2 and Table S2. Receiver operating characteristic curve analysis for progression had the shortest distance from the curve to the point (i.e., 1.0. 0.0) and were therefore used to select the cutoff value (IHC score >12). Table S1 summarizes the rate of high PGAM5 expression with respect to several standard clinicopathological features of HCC patients. Greater than 80% of the patients presented with clinical stage I–II disease in the training cohort (TMUCH), while much more patients presented with clinical stage III–IV disease in the validation cohort (SYSUCC). Correlation analysis of both the testing cohort (TMUCH patients) and validation cohort (SYSUCC patients) demonstrated that PGAM5 overexpression was positively correlated with more advanced clinical stages and tumor relapses (*p* < 0.05, Table S1). Kaplan–Meier analysis showed that PGAM5-high patients exhibited significantly shorter overall survival (OS) and progress-free survival (PFS) compared with PGAM5-low patients (OS: *p* = 0.009, log-rank test, hazard ratio (HR) = 1.819, 95% confidence interval (CI) = 1.164–2.813, Fig. 1d, Table 1.1; PFS: *p* < 0.0001, log-rank test, HR = 1.954, 95% CI = 1.358–2.811, Fig. 1e, Table 1.1). Similar results were found for the validation cohort (212 patients from SYSUCC, Fig. 1d, e, Table 1.2). Further multivariate Cox regression analysis determined that PGAM5 expression was an independent prognostic factor for poor survival in HCC patients from the TMUCH cohort (OS: *p* = 0.001, HR = 2.261, 95% CI = 1.395–3.664; PFS: *p* < 0.0001, HR = 2.335, 95% CI = 1.572–3.47, Table 2.1), and this conclusion was validated in patients from the SYSUCC cohort (OS: *p* = 0.007, HR = 1.862, 95% CI = 1.307–3.882; PFS: *p* = 0.003, HR = 2.016, 95% CI = 1.623–3.669, Table 2.2). Together, our data highlight the potential role of PGAM5 as a novel oncogenic biomarker that promotes HCC development, as high PGAM5 expression predicts poor prognoses in primary HCC patients after resection.

**Table 1.1:** Predictive variables for OS and PFS of 178 patients with HCC by univariate survival analysis (TMUCH)

Variable	OS(months)			PFS(months)	
	Cases	HR(95% CI)	*p* value	HR(95% CI)	*p* value*
Age(years)			0.129		0.397
≤55.49#	92	1.412 (0.905–2.215)		1.168 (0.815–1.674)	
>55.49	86	1.0		1.0	
Gender			0.239		0.696
Male	152	1.512 (0.789–2.615)		1.107 (0.673–1.812)	
Female	27	1.0		1.0	
Hepatitis history			0.632		0.279
Yes	131	1.132 (0.688–1.853)		1.118 (0.745–1.669)	
No	47	1.0		1.0	
AFP(ng/ml)			0.000		0.000
≤20	83	1.0		1.0	
>20	95	2.413 (1.520–3.722)		1.96 (1.375–2.832)	
Tumor size(cm)			0.004		0.001
≤5	112	1.0		1.0	
>5	66	1.906 (1.261–3.298)		1.787 (1.281–2.800)	
Tumor multiplicity			0.001		0.007
Single	145	1.0		1.0	
Multiple	35	2.242 (1.537–5.203)		1.769 (1.215–3.289)	
Stage			0.000		0.000
I–II	147	1.0		1.0	
III–IV	31	3.078 (2.693–10.92)		2.363 (1.858–5.847)	
Vascular invasion			0.005		0.008
Yes	122	2.205 (1.232–3.147)		2.118 (1.193–3.065)	
No	56	1.0		1.0	
PGAM5 expression			0.009		0.000
Low	81	1.0		1.0	
High	97	1.819 (1.164–2.813)		1.954 (1.358–2.811)	

#mean age

*Log-rank test

**Table 1.2:** Predictive variables for OS and PFS of 212 patients with HCC by univariate survival analysis (SYSUCC)

Variable	OS (months)			PFS (months)	
	Cases	HR(95% CI)	*p* value	HR(95% CI)	*p* value*
Age(years)			0.627		0.668
≤47.9#	105	1.0		1.0	
>47.9	107	1.092 (0.77–1.542)		1.061 (0.72–1.603)	
Gender			0.045		0.058
Male	174	1.651 (1.012–2.688)		1.352 (0.083–2.125)	
Female	38	1.0		1.0	
Hepatitis history			0.810		0.524
Yes	164	1.053 (0.692–1.602)		1.072 (0.059–1.633)	
No	48	1.0		1.0	
AFP(ng/ml)			0.000		0.000
≤20	67	1.0		1.0	
>20	145	2.262 (1.490–3.434)		3.389 (2.157–4.886)	
Tumor size(cm)			0.000		0.002
≤5	59	1.0		1.0	
>5	153	6.295 (3.688–10.747)		3.963 (2.597–5.559)	
Tumor multiplicity			0.000		0.001
Single	128	1.0		1.0	
Multiple	84	3.480 (2.429–4.987)		3.669 (2.582–5.139)	
Stage			0.000		0.000
I–II	88	1.0		1.0	
III–IV	124	5.393 (3.525–8.252)		4.847 (3.331–9.147)	
Vascular invasion			0.000		0.000
Yes	108	4.923 (3.331–7.322)		4.336 (3.139–6.736)	
No	104	1.0		1.0	
PGAM5 expression			0.016		0.003
Low	100	1.0		1.0	
High	112	3.165 (2.785–6.133)		4.391 (2.336–8.461)	

#mean age

*Log-rank test

**Table 2.1:** Multivariate Cox regression analysis for OS and PFS in patients with HCC (TMUCH)

Variable	OS			PFS		
	HR	95% Cl	*p* value	HR	95% Cl	*p* value
AFP, ng/ml (≤20 vs >20)	2.709	1.640–4.474	0.000	2.187	1.493–3.204	0.000
Tumor size,cm (≤5 vs >5)	1.362	0.792–2.342	0.264	1.343	0.880–2.050	0.172
Tumor multiplicity(single vs multiple)	2.267	1.333–3.856	0.003	1.635	1.037–2.578	0.034
Stage (I–II vs III–IV)	2.171	1.75–4.014	0.013	1.778	1.046–3.022	0.033
Vascular invasion	1.807	1.012–3.229	0.046	1.297	0.854–1.969	0.222
PGAM5 expression(high vs low)	1.950	1.212–3.139	0.006	2.282	1.547–3.366	0.000

*HR* hazard ratio, *CI* confidence interval

**Table 2.2:** Multivariate Cox regression analysis for OS and PFS in patients with HCC (SYSUCC)

Variable	OS			PFS		
	HR	95%Cl	*p* value	HR	95%Cl	*p* value
AFP, ng/ml (≤20 vs >20)	1.452	1.018–2.069	0.041	2.316	1.527–3.663	0.025
Tumor size, cm (≤5 vs >5)	1.621	0.888–3.053	0.165	1.337	0.726–2.176	0.216
Tumor multiplicity(single vs multiple)	1.736	0.969–42.112	0.064	2.225	1.362–3.681	0.026
Stage (I–II vs III–IV)	1.565	0.908–2.456	0.132	1.669	1.315–2.538	0.043
Vascular invasion	1.901	1.234–2.929	0.004	2.013	1.662–3.269	0.026
PGAM5 expression(positive vs negative)	1.862	1.307–3.882	0.007	2.016	1.623–3.669	0.003

*HR* hazard ratio, *CI* confidence interval

### Silencing PGAM5 inhibits HCC cell growth and induces apoptosis

**Fig. 2:**
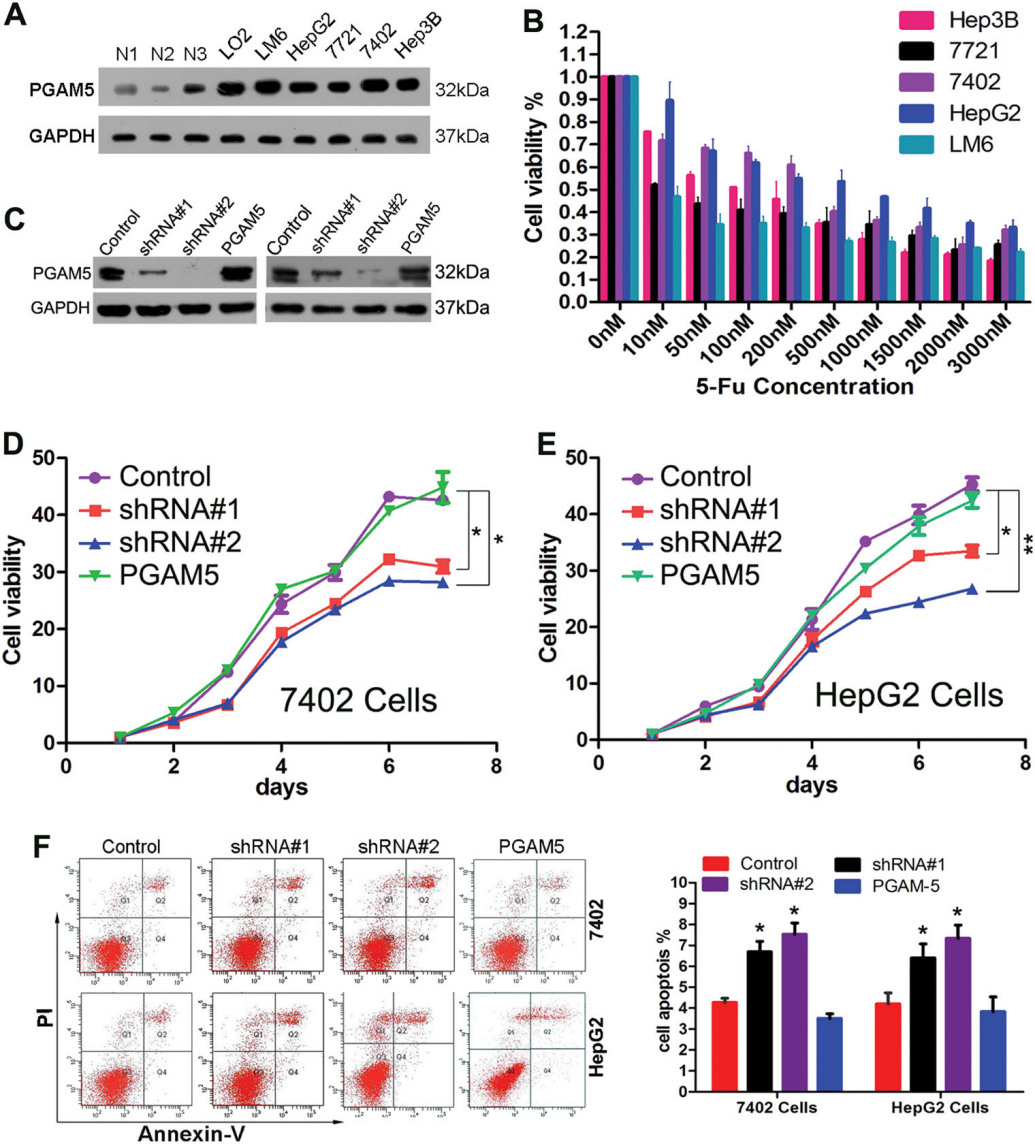
The impact of PGAM5 on HCC cell proliferation and apoptosis. **a** The levels of PGAM5 protein in 5 HCC cell lines, the immortalized liver cell line LO2 and 3 non-neoplastic hepatic tissues were examined by western blotting. **b** Cell viability of 5 HCC cell lines to 5-fliorouracil (5-Fu) were examined by CCK-8 assay. **c** Two PGAM5 shRNA (shRNA#1 and shRNA#2) were introduced to two HCC cell lines (7402 and HepG2), for stable knockdown of PGAM5 through recombinant lentiviral infection. Then, pCDH-PGAM5 lentiviral particles were transduced into above PGAM5-silened HCC cells to replenish PGAM5 expression. The levels of PGAM5 were examined by western blotting. Control = non-silencing scramble RNA sequence control; shRNA and shPGAM5 = shRNA targeting PGAM5 mRNA; PGAM5 = PGAM5-silened HCC cells with restoring the expression of PGAM5. **d**, **e** Silence of PGAM5 inhibits the proliferation of 7402 (**d**) and HepG2 (**e**) cells. The cell viabilities were detected by CCK-8 assay (*p < 0.05; **p < 0.01; Student’s *t*-test). **f** Depletion of PGAM5 increased the apoptotic rate in HCC cells. Apoptotic cells were monitored by Annexin V/propidium iodide staining and flow cytometry assays. Data represent mean values and S.E (*p *<* 0.05; Student’s *t*-test). All Data represent the mean ± S.E derived from three indivicual experiments with triplicate wells. Error bars, S.E

First, PGAM5 expression was examined by western blot in five HCC cell lines, the immortalized hepatic cell line LO2 and in three fresh adjacent non-neoplastic hepatic tissues. There were no significant differences in PGAM5 levels among these five HCC cell lines and LO2. However, PGAM5 levels were significantly higher in HCC cells than in adjacent non-tumor hepatic tissues (Fig. 2a). To further asses and confirm whether PGAM5 promotes HCC oncogenic phenotypes, we stably knocked down PGAM5 using two different PGAM5-targeted short-hairpin RNAs (shRNAs; shRNA#1 and shRNA#2) in the two HCC cell lines (7402 and HepG2) that were more resistant to 5-fluorouracil (5-Fu) (Fig. 2b). Cells transduced with corresponding control scramble shRNA served as control cells. shRNA knockdown efficiency was confirmed by western blotting (Fig. 2c), and shRNA#2 had a better silencing effect than shRNA#1. The growth and colony formation rates of PGAM5-silenced cells were significantly decreased (*p* < 0.05) compared with control cells in growth kinetic and colony formation assays (Fig. 2d, e, Supplementary Fig. S3).

To confirm that PGAM5 levels modulate HCC cell proliferation rates, we replenished PGAM5 levels in shRNA-treated 7402 and HepG2 cells via transducing a recombinant lentivirus encoding a PGAM5 construct resistant to shRNA#2 (Fig. 2c). We observed that ectopic PGAM5 overexpression rescued the survival and colony formation capacity of PGAM5-silenced HCC cells (Fig. 2d, e, Supplementary Fig. S3). To determine whether PGAM5 inhibited HCC cell apoptosis, Annexin V/propidium iodide cell apoptosis assays were performed. Compared with control cells, inhibition of PGAM5 dramatically promoted apoptosis in both 7402 and HepG2 cells (Fig. 2f). In contrast, apoptosis rates were reduced after restoring PGAM5 in PGAM5-silenced HCC cells (Fig. 2f). Together, these results indicated that PGAM5 enhanced tumor cell proliferation and inhibited apoptosis.

### Silencing PGAM5 enhances the 5-Fu sensitivity of HCC cells

**Fig. 3:**
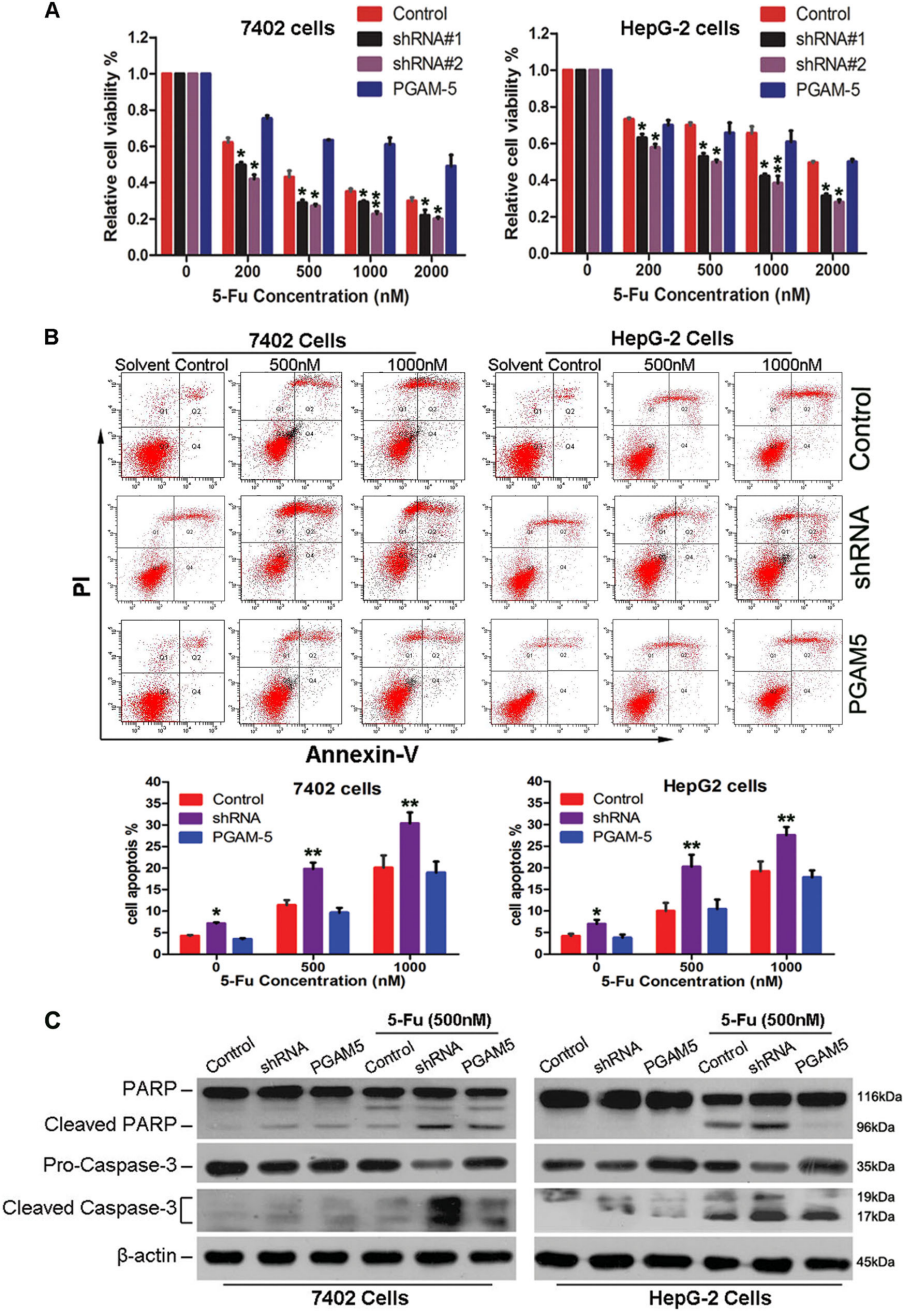
The levels of PGAM5 affected cells chemosensitivity and 5-Fu induced apoptosis in HCC cells. **a** Silence of PGAM5 inhibited HCC cell viability, whereas replenishment of PGAM5 rescued the cell viability inhibition after treatment with 5-Fu for 48 h at indicated dose. Cell viability was examined by CCK-8 assay. Data represent the mean ± SD from three individual experiments with triplicate wells (*p < 0.05; **p < 0.01, Student’s *t*-test). shRNA#2 had a better effect and was chosen for further study. **b** Silencing of PGAM5 sensitizes cells to 5-Fu induced apoptosis, whereas the apoptotic rate was attenuated after re-overexpression of PGAM5. Cell apoptotic death events were monitored by Annexin V/PI staining and flow cytometry assays. The percentage of cell apoptosis was shown as the mean ± SD from three independent experiments (*p < 0.05; **p < 0.01,Student’s *t*-test). **c** Cleaved caspase 3 and PARP induced by 5-Fu treatment were determined by Western blotting, and β-actin was used as a normalized control

Numbers of the patients with disease progression who enrolled in this study received 2–5 cycle 5-Fu-based chemotherapy. Kaplan–Meier analysis showed that patients with high PGAM5 expression exhibited chemoresistance and had significantly shorter survival times after chemotherapy compared with patients with lower PGAM5 levels (Supplementary Fig. S4). We then investigated whether PGAM5 induced HCC cell chemoresistance to 5-Fu treatment. Interesting, PGAM5-knockdown cells showed increased sensitivity to 5-Fu, as demonstrated by CCK-8 cell proliferation assays (Fig. 3a and Table S3). shRNA#2 was chosen for further study because of its superior PGAM5 silencing and chemo-sensitization effects than shRNA#1 (Figs. 2c, 3a). A significant increase in the proportion of apoptotic cells was found in PGAM5-depleted cells compared with controls under different 5-Fu concentrations (Fig. 3b). Consistently, suppressing PGAM5 induced a significant increase in cleaved PARP and caspase-3 in both HCC cell lines when the cells treated with 5-Fu (Fig. 3c). Additionally, unpaired DNA damage induced by 5-Fu, shown as γ-H2AX foci, was remarkably increased in PGAM5-knockdown HCC cells (Supplementary Fig. S5). After replenishing PGAM5 in both PGAM5-silenced HCC cell lines, the altered levels of apoptosis, and cleaved PARP and caspase-3 were restored (Fig. 3b, c). These finding suggested that increased PGAM5 expression confers chemoresistance to HCC cells may by blocking 5-Fu-induced apoptosis.

### PGAM5 confers resistance to 5-Fu-induced apoptosis in HCC cells by inhibiting BAX-cyt.C signaling via stabilizing Bcl-xL

**Fig. 4:**
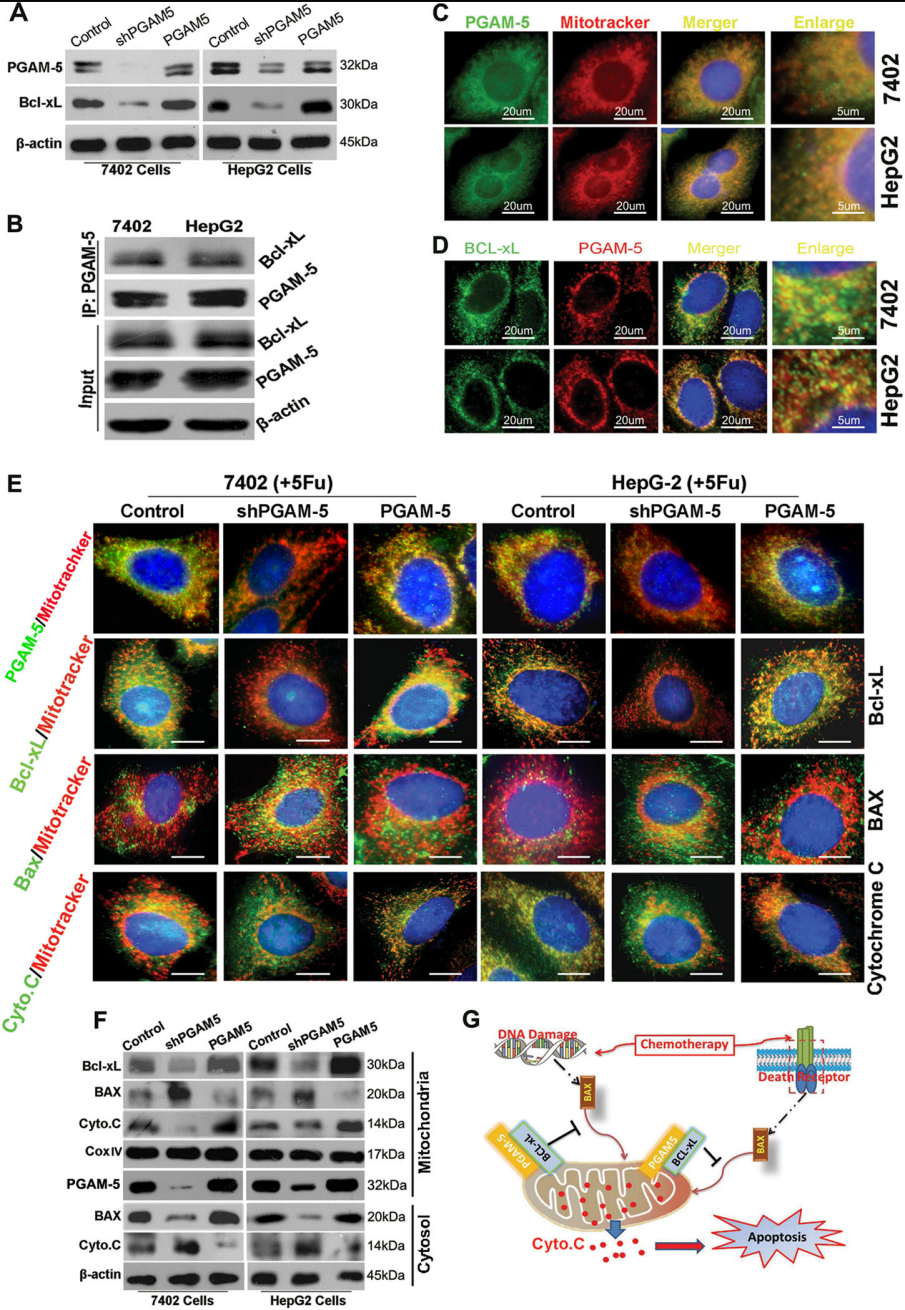
PGAM5 exerts anti-apoptotic function in HCC cells via Bcl-xL mediated apoptosis inhibition signaling pathway. **a** Depletion of PGAM5 expression substantially downregulated Bcl-xL expression in HCC cells detected by western blotting. **b** Co-immunopercipitated with PGAM5 antibody incubated shows that PGAM5 interacts with Bcl-xL in both 7402 and HepG2 cells. **c** Representative images for immune-fluorescent staining of PGAM5 and mitochondria. Nucleus was stained with DAPI (bule). Yellow dots indicate PGAM5 (green) on the mitochondria (red). **d** Immuno-staining images indicate that PGAM5 (red) and Bcl-xL (green) were co-localization (orange). **e** After 48 h treatment with 5-Fu at indicated dose, mitotracker pre-stained cells stained with Bcl-xL antibody, BAX antibody, Cyt.C antibody and PGAM-5 antibody. Representative images show that inhibition of PGAM5 decrease Bcl-xL (green) exhibition in mitochondria (upper), whereas enhances the translocation of BAX (green) from cytosol to the mitochondria (yellow dots indicated the co-localization of BAX and mitochondria, middle). In addition, silence of PGAM5 promoted the translocation of Cyt.C (green) from mitochondrial to cytosol (Yellow dots indicated the co-localization of Cyt.C and mitochondria, lower). Scale bars: 20 um. **f** Collected protein extraction respectively mitochondrial and cytosolic. Western blotting reveals that depletion of PGAM5 decrease Bcl-xL expression on mitochondrial and inhibits BAX translocation from cytoplasm to mitochondria. Proteins from the cytosolic fraction were used to determine Cyt.C release. After replenishment of PGAM5 in PGAM5-silenced 7402 and HepG2 cells, all of the altered phenotypes were recoved (**a**, **e**, and **f**). **g** Proposed schematic representation of a molecular mechanism of PGAM5, Bcl-xL, BAX, and Cyt.C in HCC chemoresistance to 5-Fu

The role of PGAM5 in HCC chemoresistance has not been established; previous reports have suggested that PGAM5 may be involved in apoptosis by interacting with Bcl-xL^31^. Interestingly, silencing PGAM5 reduced expression of the anti-apoptotic protein Bcl-xL in 7402 and HepG2 cells by western blotting (Fig. 4a). Additionally, we used an antibody against PGAM5 in an effort to co-immunoprecipitate Bcl-xL, and the results revealed a high binding affinity between PGAM5 and Bcl-xL (Fig. 4b). Detecting by immunofluorescence assay, Bcl-xL and PGAM5 showed co-localization on mitochondria (Fig. 4c, d). Interesting, Bcl-xL on mitochondria detecting by either immunofluorescence or western blotting, was significantly decreased in PGAM5-depleted HCC cells (Fig. 4e, f). To confirm that PGAM5 enhanced BCL-xL stability, HEK293 cells were transfected with shPGAM5 or vector control, and then treated with cycloheximide (CHX) to block protein synthesis for various lengths of time. BCL-xL protein levels decreased faster after CHX treatment in shPGAM5 HEK293 cells compared with vector control cells (Supplementary Fig. S6A). Additionally, the shPGAM5-induced decrease in BCL-xL was completely abolished following MG132 (a selective proteasomal inhibitor) treatment (Supplementary Fig. S6B). Moreover, immunoprecipitation assays showed that significantly more ubiquitinated BCL-xL was detected in MG132-treated shPGAM5 cells cells than MG132-treated control cells (Supplementary Fig. S6C). Together, these results demonstrated that silencing PGAM5 induced a downregulation of BCL-xL protein, which may though BCL-xL ubiquitination and proteasomal degradation. Consequently, the translocation of BAX from the cytoplasm to mitochondria was significantly increased, as was the translocation of cyt.C from mitochondria to the cytoplasm in PGAM5 knockdown cells compared with control cells; furthermore, PGAM5 rescued these cells when they were treated with 5-Fu (Figs. 4e, f). These findings suggested that inhibiting PGAM5 improved the 5-Fu chemosensitivity of HCC cells may by enhancing the translocation of BAX to mitochondria and of cyt.C to the cytosol by inducing Bcl-xL degradation (Fig. 4g).

### Silencing PGAM5 reduces tumor growth and reverses drug resistance of HCC cells in vivo

**Fig. 5:**
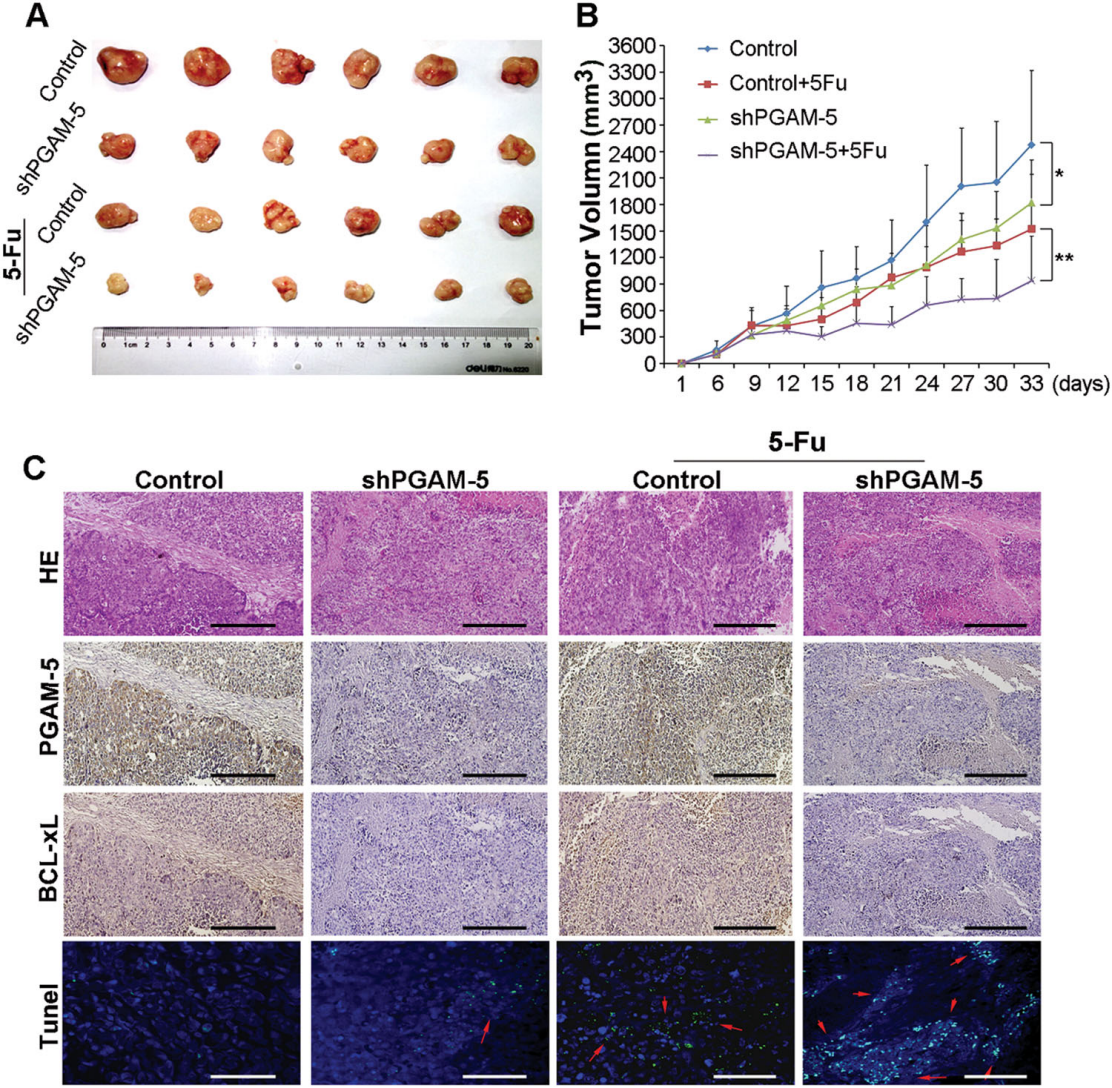
Inhibition of PGAM5 enhances the therapeutic effect of 5-Fu on HCC cell xenografts. **a**, **b** Tumor volume of xenografts was measured with calipers every 3 days for a total of 33 days. The mean tumor volume in the shPGAM5 and Control groups were 1523.09 ± 319.42 mm^3^ and 2475.59 ± 841.86 mm^3^, respectively (*n* = 6, *p < 0.05, Student’s *t*-test). After treatment of 5-Fu (Intraperitoneal injection, 10 mg/kg body weight, five times a week for 2 weeks), the mean tumor volume in the shPGAM5 group was 935.34 ± 505.58 mm^3^, which was significantly smaller than that of 1820.55 ± 779.95 mm^3^ in the Control group (*n* = 6, **p < 0.01, Student’s *t*-test). The values represent mean tumor volumes ± S.E. **c** Representative images show xenograft tumors in null mice with HepG2-Control cells and HepG2-shPGAM5 cells. H&E staining, IHC staining of PGAM5 and Bcl-xL and TUNEL assay in site were performed on sections of tumors excised from mice. Arrows indicate the TUNEL-positive staining. Scale bars: 200 um

We next sought to investigate whether inhibiting PGAM5 in HCC tumors provided a clinical advantage. To this end, we inoculated stable clones of shPGAM5 cells and control HepG2 cells into nude mice, which were treated with 5-Fu or vehicle. Tumors in mice injected with shPGAM5 cells grew significantly slower than controls. Moreover, tumors in mice that received 5-Fu plus stable shPGAM5 transfection were smaller than those treated with either 5-Fu or shPGAM5 alone (*p < 0.05; **p < 0.01, Fig. 5a, b). Analysis of PGAM5 expression in xenograft tissues by IHC confirmed that PGAM5 expression was decreased in tumors from PGAM5 knockdown cells compared with controls (Fig. 5c). As expected, silencing PGAM5 downregulated Bcl-xL expression and increased-5-Fu induced apoptosis (detected by TUNEL assay; Fig. 5c).

### Correlation between Bcl-xL levels and PGAM5 expression in HCC

**Fig. 6:**
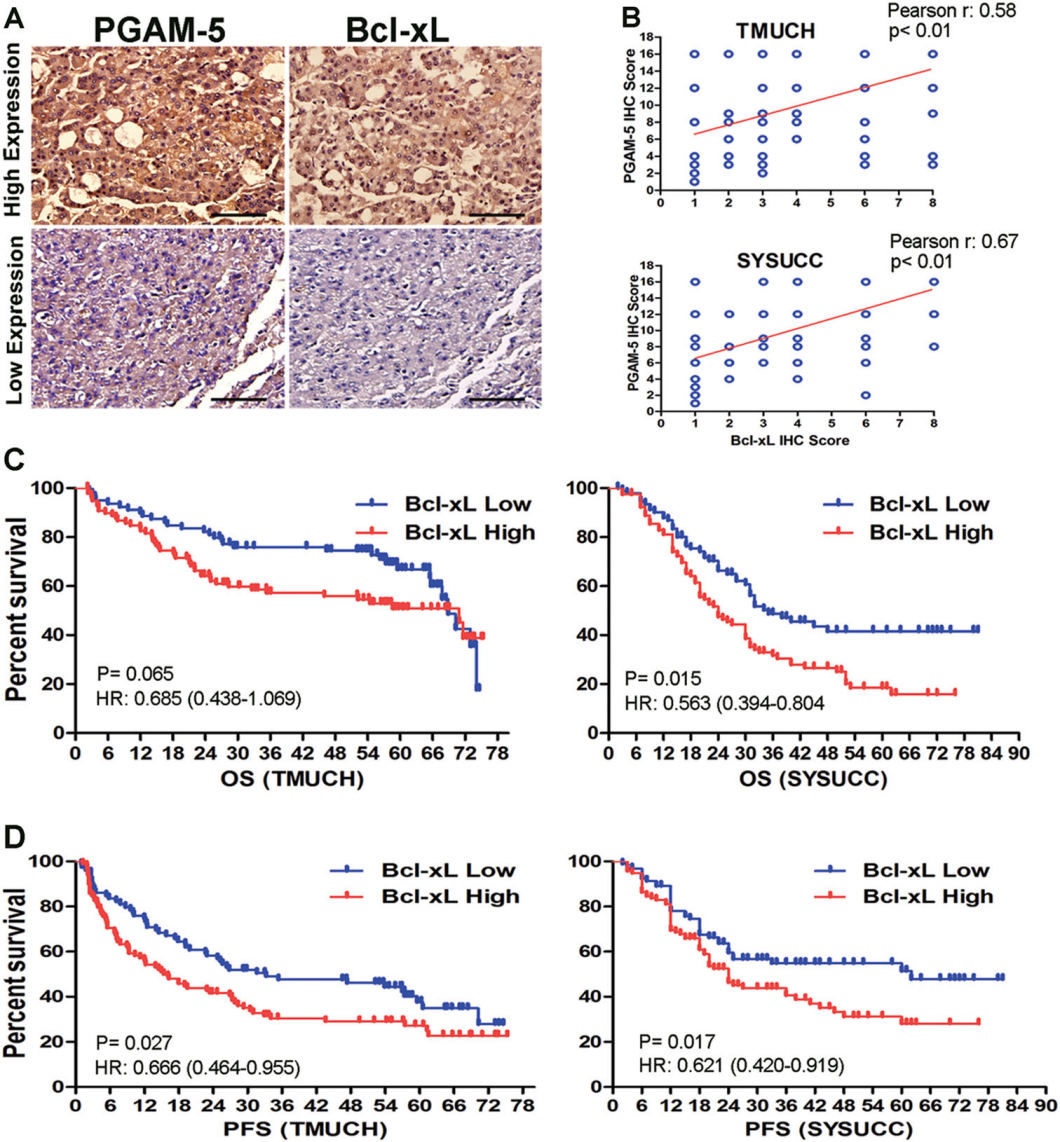
The expression of Bcl-xL was positively correlated with the PGAM5 expression level in HCC. **a** Representative immunohistochemistry images show higher expression Bcl-xL in samples with high PGAM5 levels and lower expression Bcl-xL in samples with low PGAM5 levels. Scale bars: 50 um. **b** Positively correlation between Bcl-xL and PGAM5 in HCC samples, which was determined with linear regression lines and Pearson correlation significance. (upper: the testing cohort, TMUCH; lower: the validation cohort, SYSUCC). **c**, **d** High expression of Bcl-xL is associated with poorer overall survival (left) and progression-free survival (right) in both the testing cohort (**c**) and the validation cohort (**d**)

Bcl-xL expression was further examined by IHC in the same set of specimens shown in Fig. 1. As expected, Bcl-xL levels were positively correlated with PGAM5 expression in HCC patients from both the TMUCH and SYSUCC cohorts (Pearson’s R: 0.58, *p* < 0.01 and Pearson’s R: 0.67, *p* < 0.01, respectively, Fig. 6a, b). Furthermore, higher Bcl-xL expression was also associated with poorer OS and PFS in both the learning and validation cohorts (Fig. 6c, d), which was consistent with there being higher PGAM5 levels in the same HCC specimens.

## Discussion

PGAM5 is anchored in the mitochondrial membrane and has been implicated in a diverse range of cellular activities related to controlling signal transduction pathways. To date, PGAM5 has been shown play an crucial role in the pathogenesis of liver injury^32–34^; however, little is known about the roles of PGAM5 in human carcinogenesis. In this study, we first examined PGAM5 protein expression in a series of carcinomatous and non-neoplastic human hepatic tissues. Our western blot and IHC results using an HCC TMA with complete follow-up data, clearly showed that the majority of HCC tissues examined had high PGAM5 expression levels. Subsequently, we demonstrated that overexpressed PGAM5 was significantly associated with a high risk of relapse and worse OS in HCC patients. Furthermore, our in vitro and in vivo studies showed that PGAM5 inhibition suppressed cell proliferation and tumorigenicity and promoted apoptosis by enhancing the BAX/Bcl-xL-mediated cell death signaling pathway. Taken together, our findings emphasize the fundamental roles PGAM5 in HCC, and implicate the potential of PGAM5 for prognostic predictions and therapy.

Our results are the first to clearly show that PGAM5 is frequently amplified and overexpressed in HCC tissues compared with non-tumor tissues and may be important for the acquisition of malignant HCC phenotypes. Moreover, correlation analyses revealed that high PGAM5 expression was a strong and independent predictor for poor survival in HCC patients. These observations indicate that it may be possible to use PGAM5 levels as a diagnostic tool to distinguish HCC tissues from non-malignant liver tissues or to predict the outcomes of HCC patients.

An important factor for the ineffectiveness of chemotherapy is drug-resistance, which greatly depends on the resistance to apoptosis and necrosis. PGAM5 has been proposed to function at the nexus of apoptosis and necroptosis^17,24,35,36^. We now add this body of evidence to our observations that PGAM5 depletion inhibited cell growth and increased the chemosensitivity of HCC in vivo and in vitro. Nearly every study of PGAM5 has provided evidence that it participates in early apoptotic events^37,38^. Consistently, our data revealed that PGAM5 knockdown enhanced apoptotic cell death. Furthermore, silencing PGAM5 resulted in a significant increase of proteolytically cleaved caspase3 and PARP in 5-Fu-treated cells. Additionally, TUNEL staining showed the anti-apoptotic functions of PGAM5 in vivo. Therefore, upregulating PGAM5 might enable HCC cells to overcome apoptosis and promote the development of therapeutic resistance. DNA damage mediates apoptosis by enhancing the translocation of BAX from the cytosol to mitochondria, which is a crucial mechanisms through which chemotherapy induces cell death^39,40^. In this study, we observed that silencing PGAM5 enhanced the levels of chemotherapy-induced DNA damage. PGAM5 has been identified as a component of the RIP1/RIP3-containing complexes that are formed in response to necroptosis signaling pathways^23,24^. Other studies have reported that necrotic cells are increased in PGAM5-deficient mouse embryonic fibroblasts^36^. However, Chan et al. reported that PGAM5 was dispensable for stimulus induced-necroptosis in mice. As such, there is still no consensus paradigm for how PGAM5 controls necroptosis, and there are likely context- and stimulus-dependent factors that determine whether PGAM5 acts to block or promote necroptosis^36,41–43^. Consistent with the report by Chan et al., we found no evidence to suggest that PGAM5 knockdown significantly affected the necroptosis biomarkers MLKL in HCC cell lines after 5-Fu treatment (Supplementary Fig. S7). We hypothesize that mechanisms not involving MLKL complex-mediated activation are associated with PGAM5-regulated cell death. Furthermore, the roles of PGAM5 in necroptosis may be cell- or tissue-specific^33^.

PGAM5 was recently characterized as a novel Bcl-xL binding protein that regulates hypoxia-induced mitophagy, and thus, further controlling apoptosis^44^. Bcl-xL is an anti-apoptotic protein that inhibits BAX translocation to mitochondria and cyt.C release to cytosol following the activation of downstream Caspase family and other apoptotic stimuli^45–47^. Wang et al. reported that PGAM5 is involved in the BAX-mediated apoptotic pathway in diffuse large B-cell lymphoma cells^10^. Our results from this study showed that Bcl-xL levels were decreased in PGAM5 knockdown HCC cell lines, and that the altered Bcl-xL levels were restored after replenishing PGAM5 in both PGAM5-silenced HCC cell lines. Thus, we wondered whether the protective function of PGAM5 was related to the intrinsic Bcl-xL/BAX-mediated apoptotic signaling pathway. Consistent with this hypothesis, our co-immunoprecipitation and immunofluorescence assays confirmed that PGAM5 and Bcl-xL were binding partners that were normally localized on mitochondria in the steady-state condition. Previously studies have shown that PGAM5 binds to Bcl-xL, and that this interaction involves amino acids 125 and 156 of PGAM5^16,48^. Herein, four PGAM5 deletion constructs were transfected in PGAM5-silenced 7402 cell (Supplementary Fig. S8A). Consistently, our results confirmed that PGAM5 bound to Bcl-xL via the region between amino acids 125 and 156 of PGAM5 in HCC cells using in vitro peptide binding analysis. Moreover, our results refined the key Bcl-xL-binding domain of PGAM5 to between amino acids 136 and 146 (Supplementary Fig. S8B). Additionally, our results showed that after replenishing PGAM5 in both PGAM5-silenced HCC cell lines, the enhanced chemosensitivity to 5-FU and decreased levels of Bcl-xL were restored. However, rescue experiments with the PGAM5 deletion mutant that lacked the Bcl-xL-binding domain did not result in chemoresistance to 5-FU or elevated Bcl-xL protein levels (Supplementary Fig. S8C and D). Furthermore, our CHX chase experiments and ubiquitination assays indicated that silencing PGAM5 directly induced BCL-xL degradation, possibly through the proteasome pathway. Together, our results suggested a direct interaction between PGAM5 and Bcl-xL. Blocking the interaction between PGAM5 and Bcl-xL resulted in Bcl-xL degradation and inhibited the pro-tumor functions of PGAM5.

When cells were treated with 5-Fu, PGAM5 significantly inhibited the translocation of BAX to mitochondria, blocking the release of cyt.C to the cytosol, and thus protecting cells from 5-Fu-induced apoptosis. Additionally, we observed a significant positive correlation between PGAM5 and Bcl-xL expression both in our in vivo experiments and in our large cohorts of clinical HCC tissues. The results from our HCC models strongly suggest that PGAM5 is an important upstream regulator of BAX-mediated intrinsic apoptosis, shedding new light on the mechanism of chemotherapy-induced cell death. Other studies have emphasized the fundamental roles of PGAM5 on mitophagy, mitochondrial fragmentation and necroptosis when cells face adverse conditions. These incongruous reports suggest that the functions of PGAM5 are complicated and may be specific for different tumor types or stress stimuli. Currently, it is also unclear how Bcl-xL is degraded in PGAM5-knockdown cells; thus, further studies are required.

In summary, this is the first report to describe the altered PGAM5 expression pattern in HCC and to show a potential role for PGAM5 in HCC tumorigenesis. Furthermore, functional studies of PGAM5 suggested critical roles for this mitochondrial-associated protein in apoptosis, as it regulates the Bcl-xL/BAX/cyt.C-mediated intrinsic apoptosis signaling pathway. Moreover, our results provide the basis for using PGAM5 expression as a novel predictor of disease relapse and an independent prognostic factor for HCC that would enable clinicians to identify high-risk patients that require more intensive treatment. Moreover, targeting the PGAM5 pathway may represent a new therapeutic strategy to improve survival outcomes of patients with HCC or other cancers.

## Electronic supplementary material

Supplementary Figure Legends

Supplementary and Materials and Methods

Supplementary Table 1

Supplementary Table 2

Supplementary Table 3

Supplementary Figure 1

Supplementary Figure 2

Supplementary Figure 3

Supplementary Figure 4

Supplementary Figure 5

Supplementary Figure 6

Supplementary Figure 7

Supplementary Figure 8

## References

[1] Siegel RL, Miller KD, Jemal A (2018). Cancer statistics, 2018. CA..

[2] Chen W (2016). Cancer statistics in China, 2015. CA..

[3] Llovet JM, Hernandez-Gea V (2014). Hepatocellular carcinoma: reasons for phase III failure and novel perspectives on trial design. Clin. Cancer Res..

[4] Marquardt JU, Thorgeirsson SS (2014). SnapShot: hepatocellular carcinoma. Cancer Cell..

[5] Subbotin VM (2018). Privileged portal metastasis of hepatocellular carcinoma in light of the coevolution of a visceral portal system and liver in the chordate lineage: a search for therapeutic targets. Drug Discov. Today.

[6] Finn RS (2010). Development of molecularly targeted therapies in hepatocellular carcinoma: where do we go now?. Clin. Cancer Res..

[7] Brenner D, Mak TW (2009). Mitochondrial cell death effectors. Curr. Opin. Cell Biol..

[8] Ghiotto F, Fais F, Bruno S (2010). BH3-only proteins: the death-puppeteer’s wires. Cytom. Part A J. Int. Soc. Anal. Cytol..

[9] Czabotar PE, Lessene G, Strasser A, Adams JM (2014). Control of apoptosis by the BCL-2 protein family: implications for physiology and therapy. Nat. Rev. Mol. Cell Biol..

[10] Wang P (2015). Dynamin-related protein Drp1 is required for Bax translocation to mitochondria in response to irradiation-induced apoptosis. Oncotarget.

[11] Dussmann H (2010). Single-cell quantification of Bax activation and mathematical modelling suggest pore formation on minimal mitochondrial Bax accumulation. Cell Death Differ..

[12] Chipuk JE (2008). Mechanism of apoptosis induction by inhibition of the anti-apoptotic BCL-2 proteins. Proc. Natl. Acad. Sci. U. S. A..

[13] Green DR, Galluzzi L, Kroemer G (2014). Cell biology. Metabolic control of cell death. Science.

[14] Pistritto G, Trisciuoglio D, Ceci C, Garufi A, D’Orazi G (2016). Apoptosis as anticancer mechanism: function and dysfunction of its modulators and targeted therapeutic strategies. Aging.

[15] Liu Y (2015). CHCHD2 inhibits apoptosis by interacting with Bcl-x L to regulate Bax activation. Cell Death Differ..

[16] Lo SC, Hannink M (2006). PGAM5, a Bcl-XL-interacting protein, is a novel substrate for the redox-regulated Keap1-dependent ubiquitin ligase complex. J. Biol. Chem..

[17] Takeda K (2009). Mitochondrial phosphoglycerate mutase 5 uses alternate catalytic activity as a protein serine/threonine phosphatase to activate ASK1. Proc. . Natl. Acad. Sci. U. S. A..

[18] Lo SC, Hannink M (2008). PGAM5 tethers a ternary complex containing Keap1 and Nrf2 to mitochondria. Exp. Cell Res..

[19] Niture SK, Jaiswal AK (2011). Inhibitor of Nrf2 (INrf2 or Keap1) protein degrades Bcl-xL via phosphoglycerate mutase 5 and controls cellular apoptosis. J. Biol. Chem..

[20] Lin HY (2013). Suppressor of cytokine signaling 6 (SOCS6) promotes mitochondrial fission via regulating DRP1 translocation. Cell Death Differ..

[21] Kanamaru Y, Sekine S, Ichijo H, Takeda K (2012). The phosphorylation-dependent regulation of mitochondrial proteins in stress responses. J. Signal Transduct..

[22] Chen G (2014). A regulatory signaling loop comprising the PGAM5 phosphatase and CK2 controls receptor-mediated mitophagy. Mol. Cell.

[23] Liu L, Sakakibara K, Chen Q, Okamoto K (2014). Receptor-mediated mitophagy in yeast and mammalian systems. Cell Res..

[24] Wang Z, Jiang H, Chen S, Du F, Wang X (2012). The mitochondrial phosphatase PGAM5 functions at the convergence point of multiple necrotic death pathways. Cell.

[25] Kaczmarek A, Vandenabeele P, Krysko DV (2013). Necroptosis: the release of damage-associated molecular patterns and its physiological relevance. Immunity.

[26] Wolff S, Erster S, Palacios G, Moll UM (2008). p53’s mitochondrial translocation and MOMP action is independent of Puma and Bax and severely disrupts mitochondrial membrane integrity. Cell Res..

[27] Xu W (2015). Bax-PGAM5L-Drp1 complex is required for intrinsic apoptosis execution. Oncotarget.

[28] Wu H (2014). The BCL2L1 and PGAM5 axis defines hypoxia-induced receptor-mediated mitophagy. Autophagy.

[29] Cai MY (2011). EZH2 protein: a promising immunomarker for the detection of hepatocellular carcinomas in liver needle biopsies. Gut.

[30] Kim JH (2017). A miRNA-101-3p/Bim axis as a determinant of serum deprivation-induced endothelial cell apoptosis. Cell Death Dis..

[31] Yang C (2017). Mitochondrial phosphatase PGAM5 regulates Keap1-mediated Bcl-xL degradation and controls cardiomyocyte apoptosis driven by myocardial ischemia/reperfusion injury. In Vitro Cell. Dev. Biol. Anim..

[32] He GW (2017). PGAM5-mediated programmed necrosis of hepatocytes drives acute liver injury. Gut.

[33] Ramachandran A, Jaeschke H (2017). PGAM5: a new player in immune-mediated liver injury. Gut.

[34] Hong Jeong-Min, Lee Sun-Mee (2018). Heme oxygenase-1 protects liver against ischemia/reperfusion injury via phosphoglycerate mutase family member 5-mediated mitochondrial quality control. Life Sciences.

[35] Lenhausen AM (2016). Apoptosis inducing factor binding protein PGAM5 triggers mitophagic cell death that is inhibited by the ubiquitin ligase activity of X-linked inhibitor of apoptosis. Biochemistry.

[36] Lu W (2016). Mitochondrial protein PGAM5 regulates mitophagic protection against cell necroptosis. PLoS One.

[37] Zhuang M, Guan S, Wang H, Burlingame AL, Wells JA (2013). Substrates of IAP ubiquitin ligases identified with a designed orthogonal E3 ligase, the NEDDylator. Mol. Cell.

[38] Rodins K, Gramp D, James D, Kumar S (2011). Pyogenic granuloma, port-wine stain and pregnancy. Australas. J. Dermatol..

[39] Matt S, Hofmann TG (2016). The DNA damage-induced cell death response: a roadmap to kill cancer cells. Cell. Mol. Life Sci..

[40] Catchpoole DR, Stewart BW (1993). Etoposide-induced cytotoxicity in two human T-cell leukemic lines: delayed loss of membrane permeability rather than DNA fragmentation as an indicator of programmed cell death. Cancer Res..

[41] Yang C (2017). Regulation of RIP3 by the transcription factor Sp1 and the epigenetic regulator UHRF1 modulates cancer cell necroptosis. Cell Death Dis..

[42] Moriwaki K (2016). The mitochondrial phosphatase PGAM5 is dispensable for necroptosis but promotes inflammasome activation in macrophages. J. Immunol..

[43] Safferthal C, Rohde K, Fulda S (2017). Therapeutic targeting of necroptosis by Smac mimetic bypasses apoptosis resistance in acute myeloid leukemia cells. Oncogene.

[44] Lu W (2014). Genetic deficiency of the mitochondrial protein PGAM5 causes a Parkinson’s-like movement disorder. Nat. Commun..

[45] SS R, Eastman A (2016). BCL2 inhibitors as anticancer drugs: a plethora of misleading BH3 mimetics. Mol. Cancer Ther..

[46] Del Gaizo Moore V (2007). Chronic lymphocytic leukemia requires BCL2 to sequester prodeath BIM, explaining sensitivity to BCL2 antagonist ABT-737. J. Clin. Invest..

[47] Lessene G, Czabotar PE, Colman PM (2008). BCL-2 family antagonists for cancer therapy. Nat. Rev. Drug. Discov..

[48] Hammond PW, Alpin J, Rise CE, Wright M, Kreider BL (2001). In vitro selection and characterization of Bcl-X(L)-binding proteins from a mix of tissue-specific mRNA display libraries. J. Biol. Chem..

