# Supplementary material for: High PGAM5 expression induces chemoresistance by enhancing Bcl-xL-mediated anti-apoptotic signaling and predicts poor prognosis in hepatocellular carcinoma patients
Source: Cell Death Dis. 2018 Sep 24;9(10):991. doi: 10.1038/s41419-018-1017-8 (PMC6155280; doi:10.1038/s41419-018-1017-8)
Supplement: Supplementary file 1 — Supplementary Figure Legends [file 41419_2018_1017_MOESM1_ESM.docx]

**Supplementary Figure Legends**

**Supplementary Figure 1** Representative immunohistochemistry staining in HCC tissues with different scores according to our evaluation criteria (Original magnification X400).

**Supplementary Figure 2** Receiver operation characteristic (ROC) curve analysis was employed to determine the cutoff score for the high expression of PGAM5. **(A)** In the testing cohort, progress status, Clinical stage, Tumor size and survival status implied significant statistical associations with the PGAM5 expression. There was no significant correlation between AFP levels, vascular invasion and PGAM5. **(B)** Progress status, Tumor size and survival status implied significant statistical associations with the PGAM5 expression, while Clinical stage, AFP and Vascular invasion negatively correlated with PGAM5 in validation cohort.

**Supplementary Figure 3** PGAM5 level affect HCC cells colony formation. Surviving colonies (>50cells/colony) were counted and are shown in a bar chart. Data represent the mean ± S.E (**p*< 0.05, ***p*< 0.01, Student’s t-test) derived from three individual experiments with triplicate wells. Error bars, S.E.

**Supplementary Figure 4** Association between PGAM5 expression and prognosis of patients who treated with 5-Fu based chemotherapy after progression of disease. 31 patients in the testing cohort (TMUCH) and 108 patients in validation cohort (SYSUCC) with progression of disease received 2-5 cycles of 5-Fu based chemotherapy. Kaplan-Meier analysis shows that high PGAM5 expression predicts patients’ poor survival after chemotherapy.

**Supplement Figure 5** Silencing of PGAM5 promotes 5-Fu-induced DNA damage. Shown is staining with antibodies to γH_2_AX (red) and DAPI (blue). γ-H2AX foci are used as a measure of unrepaired double strand breaks(DSB). Quantification was the average numbers of γH_2_AX foci per cell (48h after treatment). All data are derived from three individual experiments. Data represent mean values and S.E. (**p*< 0.05, ***p*< 0.01, Student’s t-test). Scale bars: 20um.

**Supplementary Figure 6** Silencing PGAM5 induced BCL-xL degradation though BCL-xL ubiquitination and proteasomal degradation. **(A)** HEK293 cells were transfected with shPGAM5 or vector control. After 24h, cells were treated with 50 ug/ml cycloheximide for different lengths of time as indicated. The levels of BCL-xL and PGAM-5 were examined by western blotting. **(B)** Levels of BCL-xL was detected by western blot in PGAM-5 silencing 7402 and HepG2 cells or control cells treated with MG132 (20uM) for 4h. **(C)**  PGAM-5 silencing 7402 and HepG2 cells were treated with MG132 (20uM) for 4h. Cell lysates were then prepared for immunoprecipitation with anti-BCL-xL antibody and ubiquitin was probed with anti-ubiquitin antibody.

**Supplementary Figure 7** PGAM5 is dispensable for 5-Fu induced necroptosis in HCC cells. Western blotting shows that PGAM5 knockdown did not alter the expression of RIP3 and pMLKL in HCC cells after treatment 5-Fu for 48h. β-actin was used as a loading control.

**Supplementary Figure 8** Bcl-xL-binding domain of PGAM5 is critical for Bcl-xL stabilization and induced chemoresistance of HCC cells to 5-Fu. (A) Schematic representation of PGAM-5 mutation domain structure. (B) Co-immunopercipitated with Flag antibody incubated shows DM-1, DM-3 and DM-4 deletion mutants failed or decreased to bind BCL-xL in PGAM-5 silencing 7402 cells. Western blot analysis of BCL-xL in whole-cell lysates from cells expressing the indicated Flag-PGAM-5 constructs. (C and D) DM-1 domain deletion mutant was unable to reverse the survival capacity and the apoptotic proportion of PGAM-5 silencing 7402 cells detected by CCK-8 assays and flow cytometry.
