# Supplementary material for: High PGAM5 expression induces chemoresistance by enhancing Bcl-xL-mediated anti-apoptotic signaling and predicts poor prognosis in hepatocellular carcinoma patients
Source: Cell Death Dis. 2018 Sep 24;9(10):991. doi: 10.1038/s41419-018-1017-8 (PMC6155280; doi:10.1038/s41419-018-1017-8)
Supplement: Supplementary file 2 — Supplementary and Materials and Methods [file 41419_2018_1017_MOESM2_ESM.docx]

**Supplementary Materials and Methods**

**The CHX experiment**

HEK293 cells were incubated with cyclohexinmide (CHX, 50ug/ml, Cell Signaling Technology, Danvers, MA, USA). to inhibit further protein synthesis. Following incubation for 0, 2, 4, 8 and 12h, cell were harvested and lysed for western blot analysis.

**Ubiquitination assay**

7402 and HepG2 cells were treated with the proteasome inhibitor MG132 (20uM, Selleck, Houston, TX, USA.) for 4H. Cells were lysated and sonicated in a cell lysis buffer (20mM Tris (pH 7.5), 150mM NaCl, 1mM EDTA, 1mM EGTA, 1% Triton X-100, 2.5mM sodium pyrophosphate, 10 mg/ml protease inhibitor cocktail) on ice. The lysates were applied to immunoprecipitation with BCL-xL antibody and immunoblotting with an ubiquitin antibody.
