# Supplementary material for: High PGAM5 expression induces chemoresistance by enhancing Bcl-xL-mediated anti-apoptotic signaling and predicts poor prognosis in hepatocellular carcinoma patients
Source: Cell Death Dis. 2018 Sep 24;9(10):991. doi: 10.1038/s41419-018-1017-8 (PMC6155280; doi:10.1038/s41419-018-1017-8)
Supplement: Supplementary file 3 — Supplementary Table 1 [file 41419_2018_1017_MOESM3_ESM.docx]

| **Table S 1.1**  Association of PGAM5 expression with patients’ clinicopathological features in primary hepatocellular carcinomas (TMUCH) | | | | | | | | | |  |
| --- | --- | --- | --- | --- | --- | --- | --- | --- | --- | --- |
|  |  |  |  |  | **PGAM5 expression** | | | | |  |
| **Variables** | |  | **Case** |  | **low** |  | **high** |  | **P Value*** |  |
| Age(years) | |  |  |  |  |  |  |  | 0.143 |  |
| ≤55.49# | |  | 92 |  | 37(40.2%) |  | 55(59.8%) |  |  |  |
| >55.49 |  |  | 86 |  | 44(51.2%) |  | 42(49.8%) |  |  |  |
| Gender |  |  |  |  |  |  |  |  | 0.904 |  |
| Male |  |  | 151 |  | 69(45.7%) |  | 82(54.3%) |  |  |  |
| Female |  |  | 27 |  | 12(44.4%) |  | 15(55.6%) |  |  |  |
| Hepatitis history | | | | | | | | | 0.372 |  |
| Yes |  |  | 131 |  | 57(43.5%) |  | 74(56.5%) |  |  |  |
| No |  |  | 47 |  | 24(51.1%) |  | 23(48.9%) |  |  |  |
| AFP(ng/ml) | |  |  |  |  |  |  |  | 0.403 |  |
| ≤20 |  |  | 83 |  | 35(42.2%) |  | 48(57.8%) |  |  |  |
| >20 |  |  | 95 |  | 46(48.4%) |  | 49(51.6%) |  |  |  |
| Tumor size(cm) | | | | | | | | | 0.209 |  |
| ≤5 |  |  | 112 |  | 55(49.1%) |  | 57(50.9%) |  |  |  |
| >5 |  |  | 66 |  | 26(39.4%) |  | 40(60.6%) |  |  |  |
| Tumor multiplicity | | | | | | | | | 0.978 |  |
| Single |  |  | 143 |  | 65(45.5%) |  | 78(54.5%) |  |  |  |
| Multiple | |  | 35 |  | 16(45.7%) |  | 19(54.3%) |  |  |  |
| Stage |  |  |  |  |  |  |  |  | 0.026 |  |
| Ⅰ |  |  | 55 |  | 19(34.5%) |  | 36(65.5%) |  |  |  |
| Ⅱ |  |  | 92 |  | 34(55.4%) |  | 46(44.6%) |  |  |  |
| Ⅲ |  |  | 21 |  | 9(42.9%) |  | 13(57.1%) |  |  |  |
| Ⅳ |  |  | 10 |  | 2(20%) |  | 8(80%) |  |  |  |
| Vascular invasion | | | | | | | | | 0.259 |  |
| Yes |  |  | 122 |  | 59(48.4%) |  | 63(51.6%) |  |  |  |
| No |  |  | 56 |  | 22(39.3%) |  | 34(60.7%) |  |  |  |
| Relapse |  |  |  |  |  |  |  |  | 0.03 |  |
| Yes |  |  | 119 |  | 45(37.8%) |  | 74(62.2%) |  |  |  |
| No |  |  | 59 |  | 36(61%) |  | 23(39%) |  |  |  |
| #mean age | |  |  |  |  |  |  |  |  |  |
| *χ^2^ test  |  |  |  |  |  |  |  |  |  |  |

| **Table S 1.2** Association of PGAM5 expression with patients’ clinicopathological features in primary hepatocellular carcinomas (SYSUCC) | | | | | | | | | | | | | | | | | | | | | | | | |  | | |  |  |
| --- | --- | --- | --- | --- | --- | --- | --- | --- | --- | --- | --- | --- | --- | --- | --- | --- | --- | --- | --- | --- | --- | --- | --- | --- | --- | --- | --- | --- | --- |
|  |  |  | |  | | |  | | | **PGAM5 expression** | | | | | | | | | | | | | | |  | | |  |  |
| **Variables** | |  | | **Case** | | |  | | | **low** | | |  | | | **high** | | |  | | | **P Value*** | | |  | | |  |  |
| Age(years) | |  | |  | | |  | | |  | | |  | | |  | | |  | | | 0.271 | | |  | | |  |  |
| ≤47.9# | |  | | 105 | | |  | | | 50(47.6%) | | |  | | | 55(52.4%) | | |  | | |  | | |  | | |  |  |
| >47.9 |  |  | | 107 | | |  | | | 50(46.7%) | | |  | | | 57(53.3%) | | |  | | |  | | |  | | |  |  |
| Gender |  |  | |  | | |  | | |  | | |  | | |  | | |  | | | 0.443 | | |  | | |  |  |
| Male |  |  | | 174 | | |  | | | 83(47.7%) | | |  | | | 91(52.3%) | | |  | | |  | | |  | | |  |  |
| Female |  |  | | 38 | | |  | | | 17(44.7%) | | |  | | | 21(55.3%) | | |  | | |  | | |  | | |  |  |
| Hepatitis history | | | | | | | | | | | | | | | | | | | | | | 0.772 | | |  | | |  |  |
| Yes |  |  | | 164 | | |  | | | 79(48.2%) | | |  | | | 85(51.8%) | | |  | | |  | | |  | | |  |  |
| No |  |  | | 48 | | |  | | | 21(43.7%) | | |  | | | 27(56.3%) | | |  | | |  | | |  | | |  |  |
| AFP(ng/ml) | |  | |  | | |  | | |  | | |  | | |  | | |  | | | 0.751 | | |  | | |  |  |
| ≤20 |  |  | | 67 | | |  | | | 33(49.3%) | | |  | | | 34(50.7%) | | |  | | |  | | |  | | |  |  |
| >20 |  |  | | 145 | | |  | | | 67(46.2%) | | |  | | | 78(53.8%) | | |  | | |  | | |  | | |  |  |
| Tumor size(cm) | | | | | | | | | | | | | | | | | | | | | | 0.637 | | |  | | |  |  |
| ≤5 |  |  | | 59 | | |  | | | 27(45.8%) | | |  | | | 32(54.2%) | | |  | | |  | | |  | | |  |  |
| >5 |  |  | | 153 | | |  | | | 73(47.7%) | | |  | | | 80(52.3%) | | |  | | |  | | |  | | |  |  |
| Tumor multiplicity | | | | | | | | | | | | | | | | | | | | | | 0.527 | | |  | | |  |  |
| Single |  |  | | 128 | | |  | | | 58(45.3%) | | |  | | | 70(54.7%) | | |  | | |  | | |  | | |  |  |
| Multiple | |  | | 84 | | |  | | | 42(50.0%) | | |  | | | 42(50.0%) | | |  | | |  | | |  | | |  |  |
| Stage |  |  | |  | | |  | | |  | | |  | | |  | | |  | | | 0.011 | | |  | | |  |  |
| Ⅰ |  |  | | 28 | | |  | | | 17(60.7%) | | |  | | | 11(39.3%) | | |  | | |  | | |  | | |  |  |
| Ⅱ |  |  | | 60 | | |  | | | 37(61.7%) | | |  | | | 23(38.3%) | | |  | | |  | | |  | | |  |  |
| Ⅲ |  |  | | 97 | | |  | | | 39(40.2%) | | |  | | | 58(59.8%) | | |  | | |  | | |  | | |  |  |
| Ⅳ |  |  | | 27 | | |  | | | 7(25.9%) | | |  | | | 20(74.1%) | | |  | | |  | | |  | | |  |  |
| Vascular invasion | | | | | | | | | | | | | | | | | | | | | | 0.047 | | |  | | |  |  |
| Yes |  | |  | | | 108 | | |  | | | 65(60.2%) | | |  | | | 43(39.8%) | | |  | | |  | | |  | | |
| No |  | |  | | | 104 | | |  | | | 35(33.7%) | | |  | | | 69(66.3%) | | |  | | |  | | |  | | |
| Relapse |  | |  | | |  | | |  | | |  | | |  | | |  | | |  | | | 0.016 | | |  | | |
| Yes |  | |  | | | 105 | | |  | | | 34(32.4%) | | |  | | | 71(67.6%) | | |  | | |  | | |  | | |
| No |  | |  | | | 107 | | |  | | | 66(61.7%) | | |  | | | 41(38.3%) | | |  | | |  | | |  | | |
| #mean age | |  | | |  | | |  | | |  | | |  | | |  | | |  | | |  | | |  | | |  |
| *χ^2^ test  |  | |  | | |  | | |  | | |  | | |  | | |  | | |  | | |  | | |  | | |
