# Supplementary material for: High PGAM5 expression induces chemoresistance by enhancing Bcl-xL-mediated anti-apoptotic signaling and predicts poor prognosis in hepatocellular carcinoma patients
Source: Cell Death Dis. 2018 Sep 24;9(10):991. doi: 10.1038/s41419-018-1017-8 (PMC6155280; doi:10.1038/s41419-018-1017-8)
Supplement: Supplementary file 4 — Supplementary Table 2 [file 41419_2018_1017_MOESM4_ESM.docx]

|  | **TMUCH*** | | | | **SYSUCC^** | | |
| --- | --- | --- | --- | --- | --- | --- | --- |
| **Factors** | **ROC^#^** | **P value** | **Cut-off value** | **ROC** | | **P value** | **Cut-off value** |
| Progress status, yes or no | 0.675 | 0.002 | 12 | 0.610 | | 0.013 | 9/12 |
| Clinical stage,Ⅰ/Ⅱ vs Ⅲ/Ⅳ | 0.676 | 0.008 | 12 | 0.555 | | 0.176 |  |
| Tumor Size (cm), ≤ 5 VS > 5 | 0.632 | 0.012 | 12 | 0.588 | | 0.048 | 9/12 |
| Survival status, Live VS death | 0.617 | 0.018 | 12 | 0.675 | | 0.007 | 12 |
| AFP(ng/ml), ≤20 VS > 20 | 0.465 | 0.419 |  | 0.436 | | 0.108 |  |
| Vascular invasion, yes VS no | 0.553 | 0.268 |  | 0.535 | | 0.373 |  |

Table S2. Receiver operating characteristic curve analysis for PGAM5 expresson with respect to patients’ key clinicopathological features.

*Tianjin medical university cancer hospital; **^**Sun Yat-Sen University Cancer Center; #receiver operating characteristic curve
