# Supplementary material for: High PGAM5 expression induces chemoresistance by enhancing Bcl-xL-mediated anti-apoptotic signaling and predicts poor prognosis in hepatocellular carcinoma patients
Source: Cell Death Dis. 2018 Sep 24;9(10):991. doi: 10.1038/s41419-018-1017-8 (PMC6155280; doi:10.1038/s41419-018-1017-8)
Supplement: Supplementary file 5 — Supplementary Table 3 [file 41419_2018_1017_MOESM5_ESM.docx]

**Table S3** The IC50 of 7402 and HepG2 cell lines to 5-fluorouracil (5-Fu)

Statistical differences compared with the control group (7402/HepG2) is given as **p* < 0.05.
