# Supplementary figures and images for: High PGAM5 expression induces chemoresistance by enhancing Bcl-xL-mediated anti-apoptotic signaling and predicts poor prognosis in hepatocellular carcinoma patients
Source: Cell Death Dis. 2018 Sep 24;9(10):991. doi: 10.1038/s41419-018-1017-8 (PMC6155280; doi:10.1038/s41419-018-1017-8)

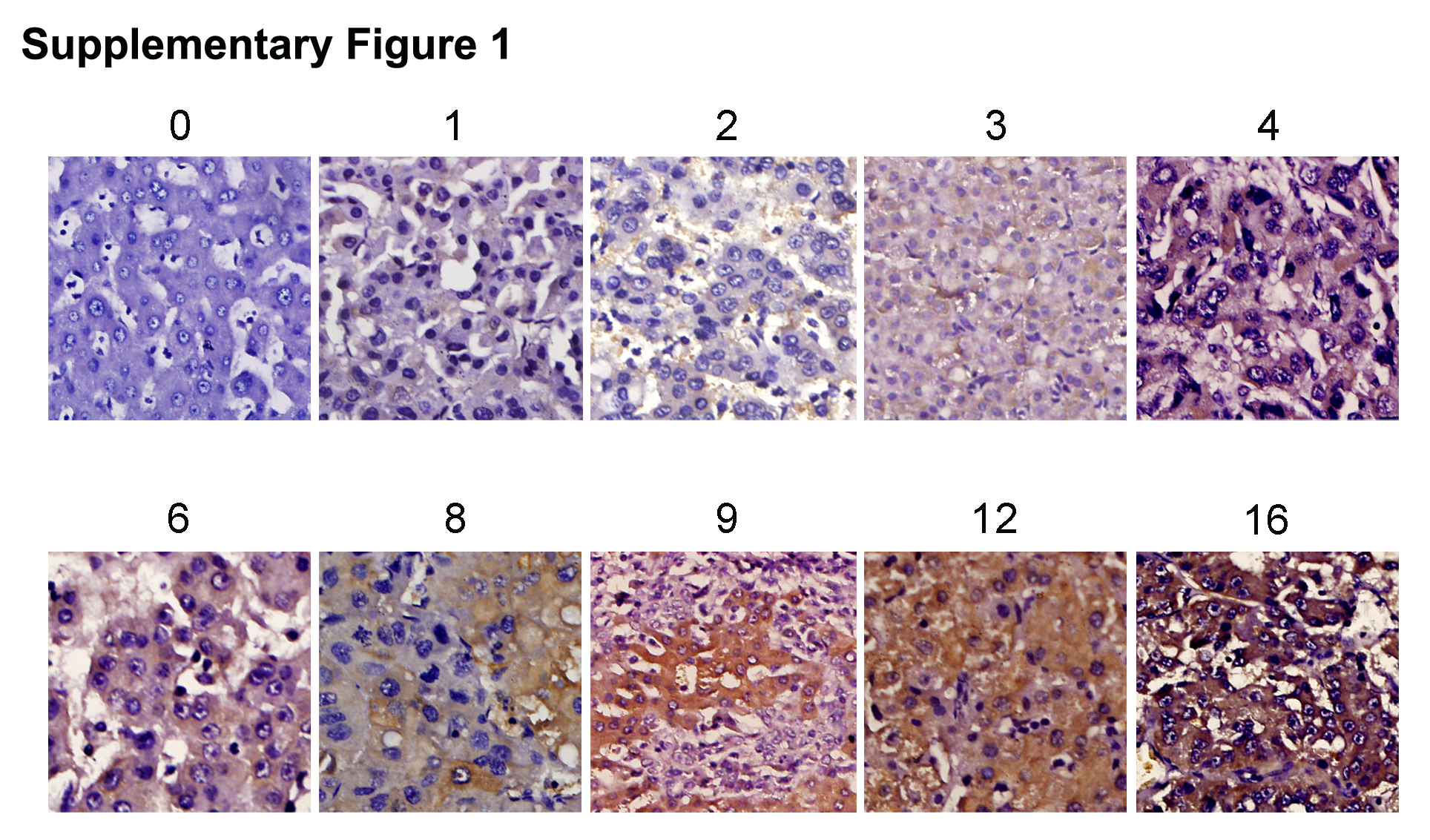

Supplement: Supplementary file 6 — Supplementary Figure 1 [file 41419_2018_1017_MOESM6_ESM.tif]

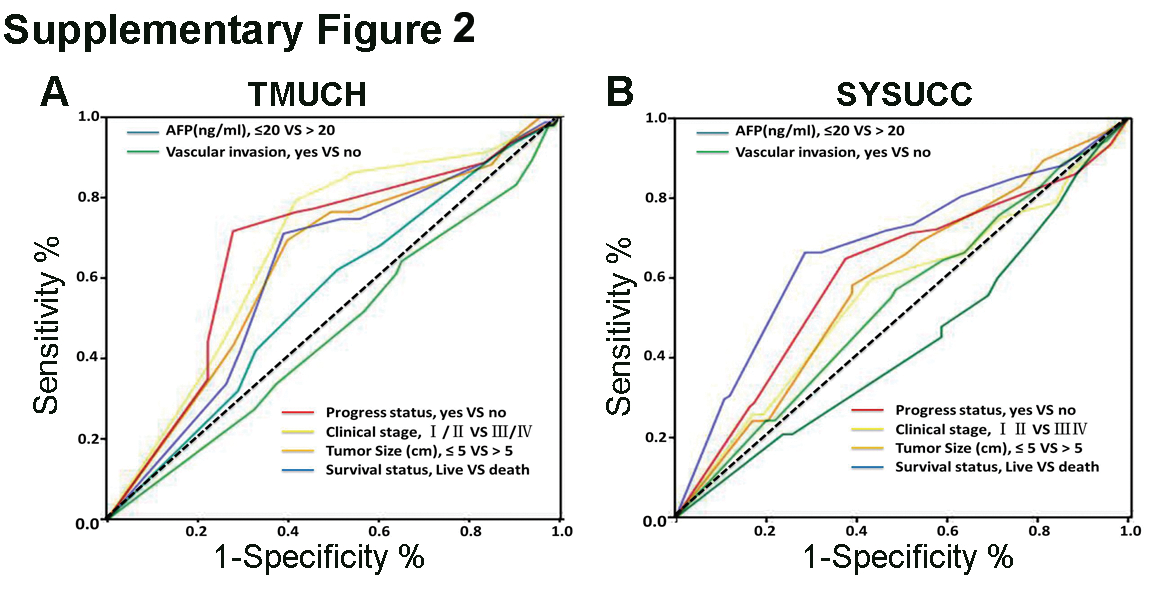

Supplement: Supplementary file 7 — Supplementary Figure 2 [file 41419_2018_1017_MOESM7_ESM.tif]

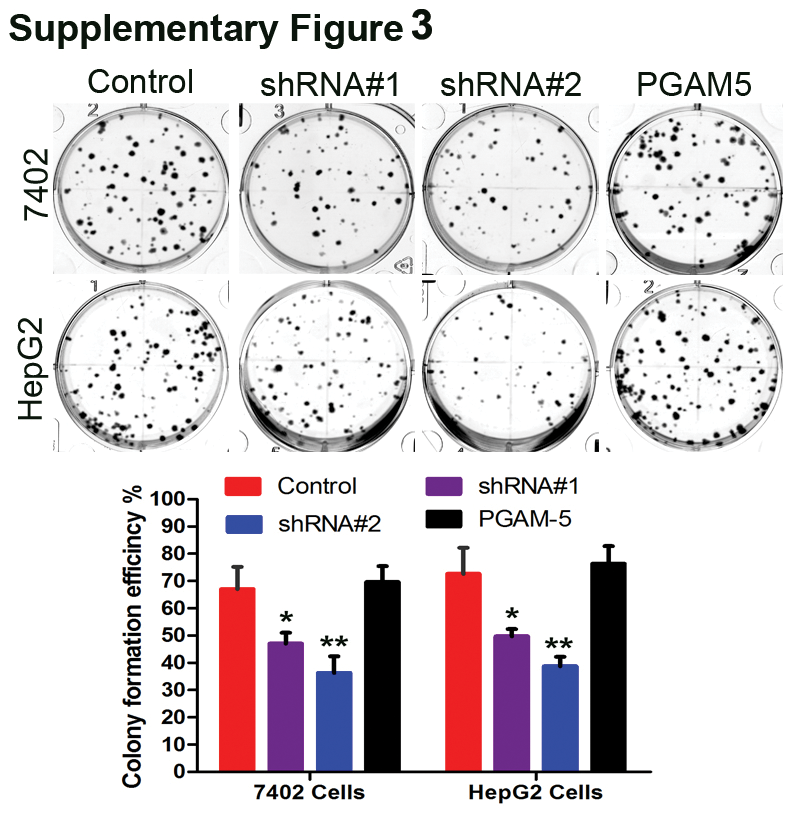

Supplement: Supplementary file 8 — Supplementary Figure 3 [file 41419_2018_1017_MOESM8_ESM.tif]

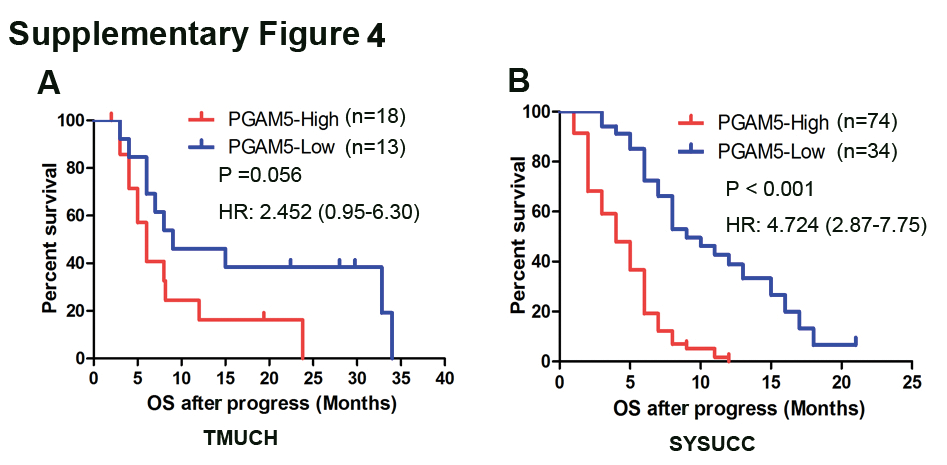

Supplement: Supplementary file 9 — Supplementary Figure 4 [file 41419_2018_1017_MOESM9_ESM.tif]

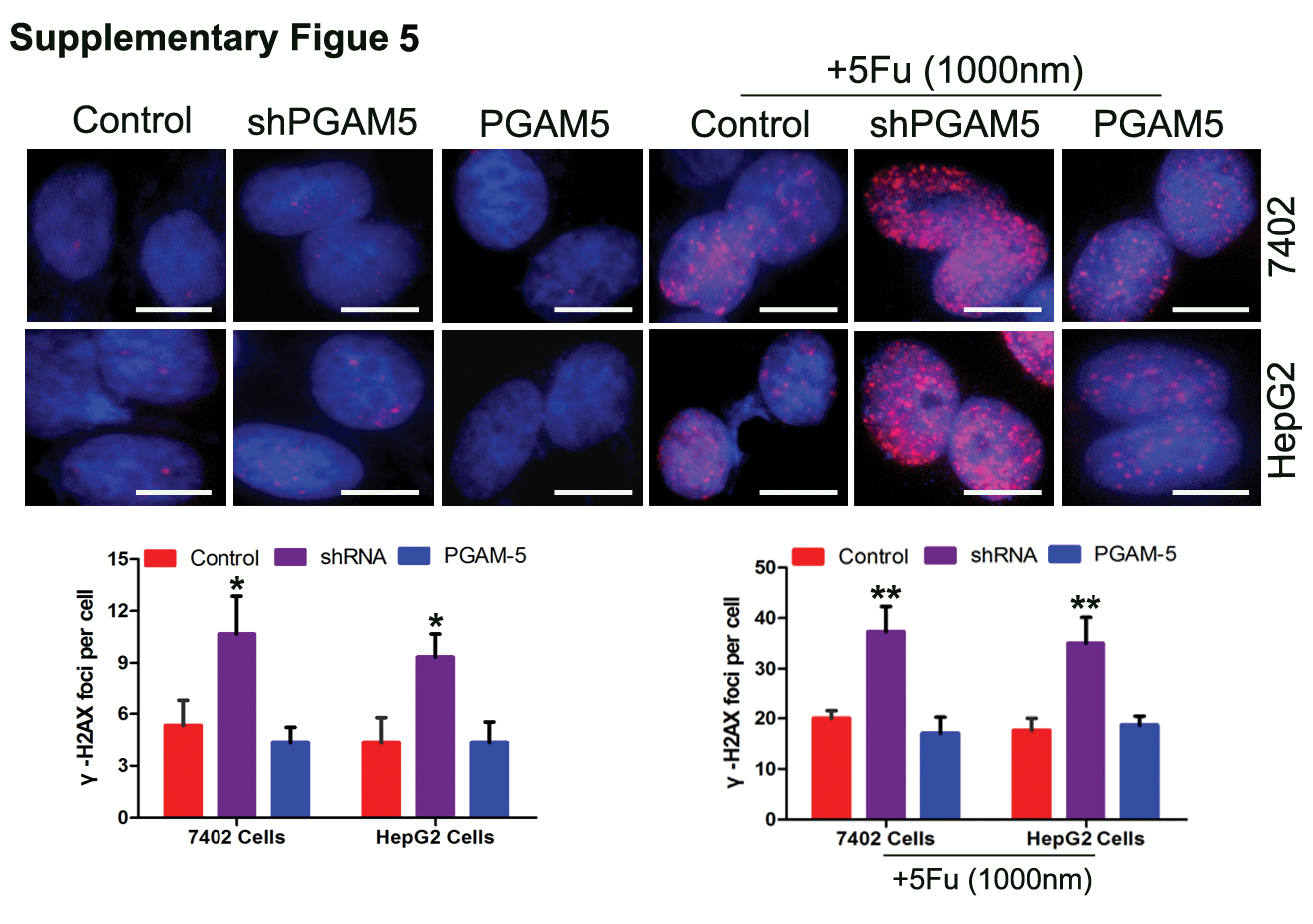

Supplement: Supplementary file 10 — Supplementary Figure 5 [file 41419_2018_1017_MOESM10_ESM.tif]

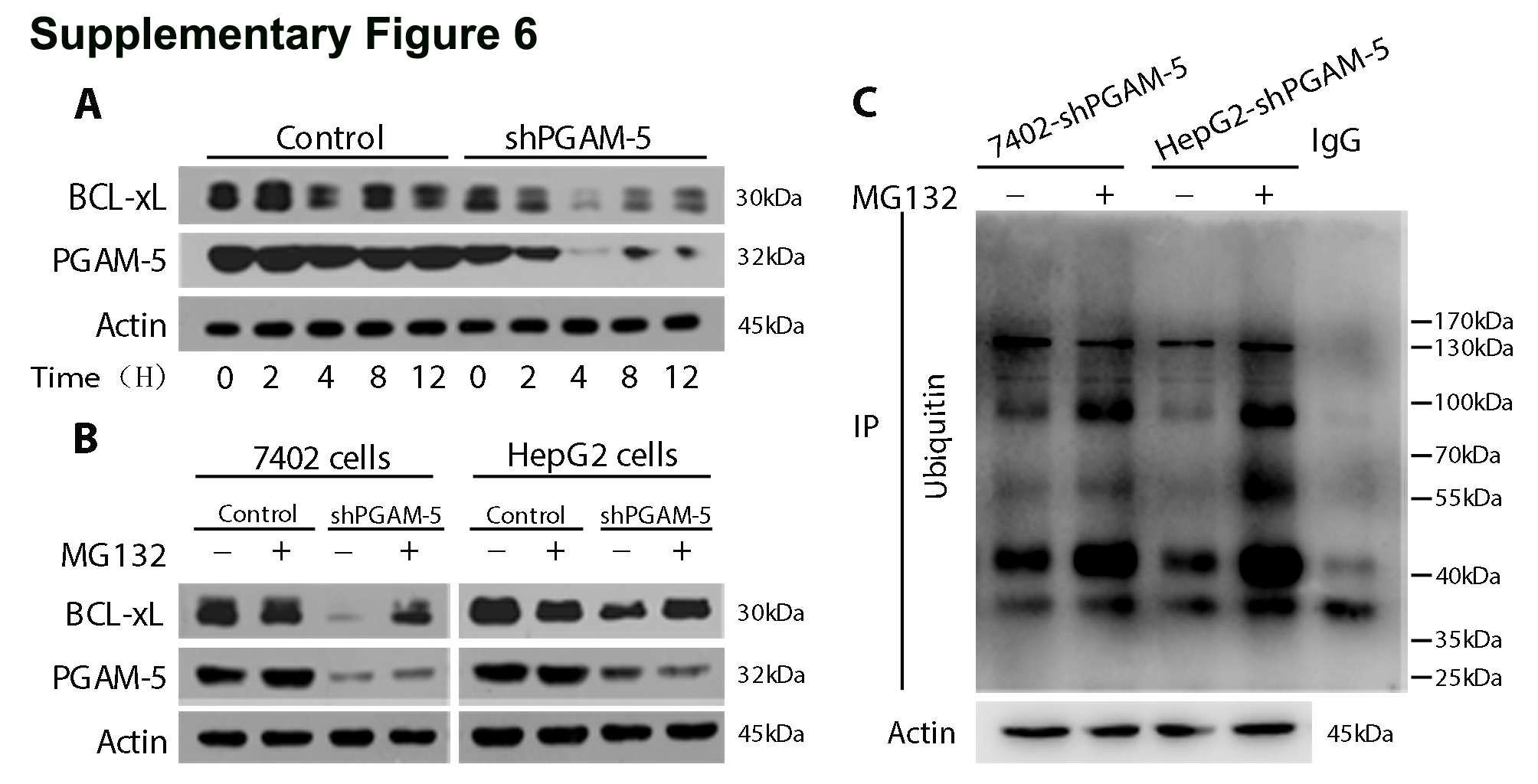

Supplement: Supplementary file 11 — Supplementary Figure 6 [file 41419_2018_1017_MOESM11_ESM.tif]

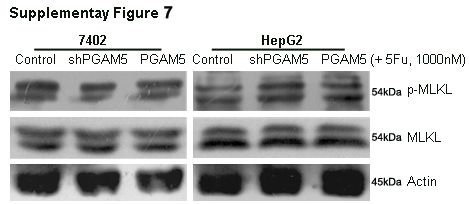

Supplement: Supplementary file 12 — Supplementary Figure 7 [file 41419_2018_1017_MOESM12_ESM.tif]

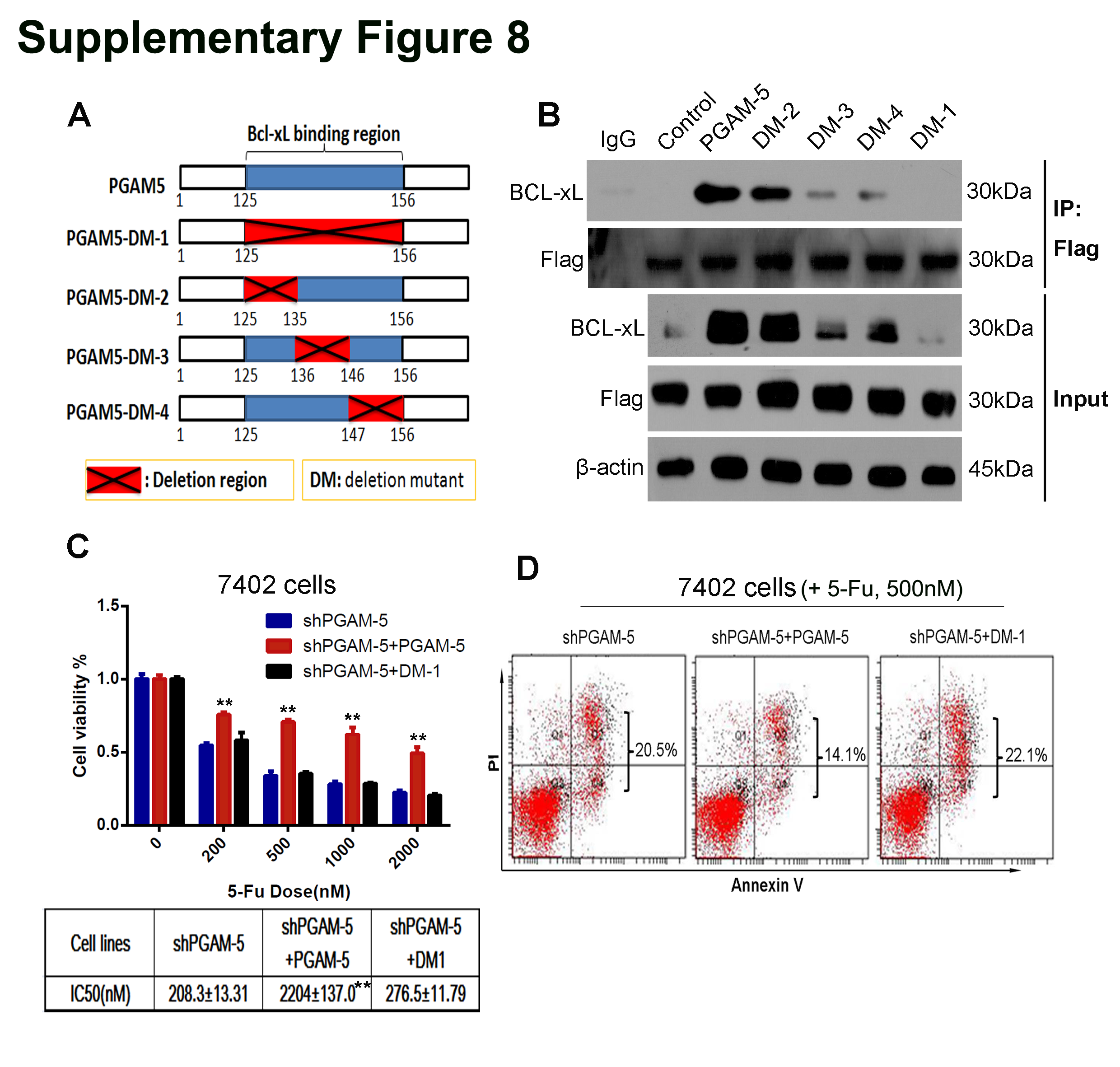

Supplement: Supplementary file 13 — Supplementary Figure 8 [file 41419_2018_1017_MOESM13_ESM.tif]
